# Annotation of uORFs in the OMIM genes allows to reveal pathogenic variants in 5′UTRs

**DOI:** 10.1093/nar/gkac1247

**Published:** 2023-01-18

**Authors:** Alexandra Filatova, Ivan Reveguk, Maria Piatkova, Daria Bessonova, Olga Kuziakova, Victoria Demakova, Alexander Romanishin, Veniamin Fishman, Yerzhan Imanmalik, Nikolay Chekanov, Rostislav Skitchenko, Yury Barbitoff, Olga Kardymon, Mikhail Skoblov

**Affiliations:** Research Centre for Medical Genetics, Moscow, Russia; Laboratoire de Biologie Structurale de la Cellule, École Polytechnique, Paris, France; Institute of Chemistry, Far Eastern Branch of the Russian Academy of Sciences, Vladivostok, Russia; Institute of high technologies and advanced materials, Far Eastern Federal University, Vladivostok, Russia; Medical Center, Far Eastern Federal University, Vladivostok, Russia; Institute of Life Sciences and Biomedicine, Far Eastern Federal University, Vladivostok, Russia; Medical Center, Far Eastern Federal University, Vladivostok, Russia; Institute of Life Sciences and Biomedicine, Far Eastern Federal University, Vladivostok, Russia; Institute of Life Sciences, Immanuel Kant Baltic Federal University, Kaliningrad, Russia; Artificial Intelligence Research Institute, Moscow, Russia; Molecular Mechanisms of Ontogenesis, Institute of Cytology and Genetics SB RAS, Novosibirsk, Russia; Artificial Intelligence Research Institute, Moscow, Russia; Artificial Intelligence Research Institute, Moscow, Russia; Computer Technologies Laboratory, ITMO University, Saint Petersburg, Russia; Bioinformatics Institute, St. Petersburg, Russia; Department of Genomic Medicine, D.O. Ott Research Institute of Obstetrics, Gynaecology, and Reproductology, St. Petersburg, Russia; Dpt. of Genetics and Biotechnology, St. Petersburg State University, St. Petersburg, Russia; Artificial Intelligence Research Institute, Moscow, Russia; Research Centre for Medical Genetics, Moscow, Russia

## Abstract

An increasing number of studies emphasize the role of non-coding variants in the development of hereditary diseases. However, the interpretation of such variants in clinical genetic testing still remains a critical challenge due to poor knowledge of their pathogenicity mechanisms. It was previously shown that variants in 5′-untranslated regions (5′UTRs) can lead to hereditary diseases due to disruption of upstream open reading frames (uORFs). Here, we performed a manual annotation of upstream translation initiation sites (TISs) in human disease-associated genes from the OMIM database and revealed ∼4.7 thousand of TISs related to uORFs. We compared our TISs with the previous studies and provided a list of ‘high confidence’ uORFs. Using a luciferase assay, we experimentally validated the translation of uORFs in the *ETFDH*, *PAX9*, *MAST1*, *HTT*, *TTN*,*GLI2* and *COL2A1* genes, as well as existence of N-terminal CDS extension in the *ZIC2* gene. Besides, we created a tool to annotate the effects of genetic variants located in uORFs. We revealed the variants from the HGMD and ClinVar databases that disrupt uORFs and thereby could lead to Mendelian disorders. We also showed that the distribution of uORFs-affecting variants differs between pathogenic and population variants. Finally, drawing on manually curated data, we developed a machine-learning algorithm that allows us to predict the TISs in other human genes.

## INTRODUCTION

Despite the expanding use of the Next-Generation Sequencing (NGS) technology for molecular diagnostics of Mendelian disorders, ∼50–75% of the patients do not receive a genetic diagnosis after DNA testing (1). One of the reasons for this is that the interpretation of disease-causing variants generally focuses on the coding part of the genes. The detection and prioritization of non-coding variants still remains a critical challenge due to poor knowledge of their possible pathogenicity mechanisms.

Upstream open reading frames (uORFs) are ORFs located in the 5′ untranslated regions (5′UTRs) of protein-coding genes. uORFs can influence the translation of the gene coding sequences (CDS) by numerous mechanisms, including translation reinitiation, leaky‐scanning, and ribosome‐stalling (2,3). Several previous studies revealed nucleotide variants in 5′UTRs that affect uORFs and lead to hereditary diseases and malignancies. These variants may create a new ATG codon, resulting in a new uORF (4–7), or may disrupt the existing uORFs (8–11). Moreover, Whiffin *et al.* have previously shown that both of these types of 5′UTR variants are under strong negative selection (12). Thus, we suggest that there should be many more pathogenic variants in the 5′UTRs, and current routine diagnostic algorithms miss them.

Previous studies have described a large number of uORFs in human genes. Using a RibORF algorithm to analyze ribosome profiling (Ribo-seq) data of two human cell lines, Ji *et al.* found ∼8000 translated uORFs in ∼35% of mRNA of coding genes (13). Later McGillivray *et al.* created a comprehensive catalog of predicted human uORFs (14). Using their own machine learning algorithm, the authors analyzed Ribo-seq and mass-spectrometry data from the previous studies (15–18) and identified ∼189 000 likely active uORFs in 11 171 human genes. In another study, Scholz *et al.* provided 1933 uORFs in 1703 genes based on 35 data sets from nine human ribosome profiling series (19). Such a variety of data makes it difficult to use them to interpret nucleotide variants identified by clinical genetic testing. A very recent Phase I study of standardized computational annotation of translated open reading frames (20) confirms the need for a high-quality uORFs catalog.

In recent years, a large amount of Ribo-seq data has been accumulated. User-friendly web browsers were created to allow researchers to manually explore publicly available data. GWIPS-viz (21) and Trips-Viz (22) browsers provide data about more than 40 ribosomal profiling studies in >20 human cell lines. Along with datasets representing footprints of elongating ribosomes, GWIPS-viz provides datasets with enrichment footprints deriving from initiating ribosomes that can facilitate the identification of the translation initiation sites (TISs).

In the present study, we performed a manual annotation of uORFs in the 5'UTR of human genes included in the OMIM (Online Mendelian Inheritance in Man) database. For this, we used data presented in the GWIPS-viz and Trips-Viz browsers, as well as the analysis of predicted ORFs and Kozak sequence strength in tandem with mRNA-seq data and investigation of transcription start sites (23). We identified ∼5.2 thousand additional upstream TISs related to both ATG and non-ATG start codons. Annotated TISs belong to different types of ORFs: uORFs that overlap and do not overlap with the downstream gene CDSs (∼4.7 thousand) and alternative TISs leading to extension or truncation of the reference coding sequence. We compared our TISs with the previous studies and experimentally tested ten cases using luciferase assay. Besides, we implemented a tool to annotate the effects of genetic variants located in uORFs. We used this tool to evaluate the effects of known pathogenic variants from the Human Genome Mutation Database (HGMD) (24) and ClinVar (25) as well as variants present in the Genome Aggregation Database (gnomAD) genomes (26). For previously described pathogenic variant c.-75A >G in the *ETFDH* gene we performed luciferase experiments and showed that this variant reduces the translation of the main gene CDS due to the disruption of uORF stop codon.

Moreover, we used our manually curated dataset to create a generalized model. We attempted to build on recent successes in biological sequence modeling tasks (27–29) made mainly possible thanks to various incarnations of the transformer architecture (30). We discovered that the neural network's usage suffered from overfitting and, in general, showed a limited performance. On the other hand, a simpler model, based on the gradient-boosted decision trees of XGBoost, outperformed neural network-based architectures. Hence, we trained an XGBoost model on a manually curated dataset and applied it for TISs’ prediction within 5′UTRs of human protein-coding genes not included in manual annotation, as well as in human lncRNA genes.

## MATERIALS AND METHODS

### Data and tools for manual upstream translation initiation site curation

We manually annotated upstream translation initiation sites (TISs) for genes associated with Mendelian disorders from the OMIM database (last date of access July 2021) (31). The data curation process relied on the publicly available Ribo-seq and RNA-seq data from the GWIPS-viz (21) and Trips-Viz (22) browsers (last date of access September 2022). Transcription start sites (TSSs) were identified using FANTOM5 CAGE data (23).

The prediction of the Kozak-sequence score was carried out with the AltTranslationInitiation tool (https://github.com/ewallace/AltTranslationInitiation) using nucleotide frequencies from Grzegorski *et al.* (32). We predicted the Kozak score for transcripts from Ensembl Genes 103 (GRCh38.p13) database. For each transcript, we extracted sequences of 5′UTR along with the 100 bp downstream and 500 upstream and performed Kozak score predictions for ATG and near-cognate codons (TTG, GTG, CTG, AAG, AGG, ACG, ATA, ATT, ATC). Our Kozak sсore prediction tool was modified to ignore the strength of the start codon itself. Thus, our Kozak score reflects only the surrounding context of the analyzed start codon but not its own translation initiation potential.

We obtained the lists of likely active human uORFs from the studies by McGillivray *et al.* (14), Ji *et al.* (13) and Scholz *et al.* (19). Besides, we used mouse uORF list from the Wang *et al.* study (33). We generated BED files with these uORFs using in-house software and re-mapped all of them to the Human hg38 genome by NCBI Genome Remapping Service.

Manually curated additional translation initiation sites (TISs) were converted to open reading frames using an in-house tool and relying on the human gene annotation from HGMD subset of NCBI RefSeq genes (109.20211119 (2021-11-23)). Next, we manually re-checked that the uORF alignment was correct, and that HGMD mRNA isoform matched to RNA-seq and CAGE data. In some ambiguous cases, the most appropriate mRNA isoforms for ORF generation were manually selected. After that, our tool was run again on the updated list of mRNA isoforms. The final ORF list is presented in Supplementary Data S1 (ATG-started ORFs) and S2 (non-ATG-started ORFs). In addition, an updated online version of this list in the BED format is available at https://doi.org/10.5281/zenodo.7435228. Genomic coordinates of ORFs are given according Human genome assembly hg38. Characterization of the annotated TISs groups, as well as analysis of their Kozak scores and translation initiation signal, was performed in R software, and the Wilcoxon two-sided Rank Sum test was used for statistical analysis (*P*-values were corrected with the Holm–Bonferroni correction method).

### Plasmids

For cloning, we used the psiCHECK-2 vector (Promega, USA). At first, we replaced the vector-derived ATG start codon of the *hRluc* with TTG, thereby preventing its background translation. To obtain the wild-type plasmids for each gene of interest, we amplified 5′UTR with a few first codons using genomic DNA (for single exon 5′UTRs) or complementary DNA of a healthy donor's primary skin fibroblasts (for multiple exons 5′UTRs). To determine the exact sequences for cloning, we used isoforms listed in Human Genome Mutation Database (HGMD Professional v2021.3) as reference isoforms for most of the studied genes. If the 5′end of HGMD isoform did not coincide with the major FANTOM5 CAGE transcription start site, we used the isoform reflecting the CAGE-derived 5′end. Thus, we used the following isoforms: NM_002111.8 (*HTT*), NM_001844.5 (*COL2A1*), NM_133378.4 (*TTN*), NM_005270.5 (*GLI2*), NM_001014987.2 (*LAT*), NM_001122821.2 (*SET*), NM_001372076.1 (*PAX9*), NM_014975.3 (*MAST1*), NM_014268.4 (*MAPRE2*), NM_007129.5 (*ZIC2*), NM_004453.4 (*ETFDH*). Next, we cloned the obtained PCR products into a modified psiCHECK-2 vector using Gibson Assembly Master Mix (NEB, USA).

To introduce the studied mutations into wild-type plasmids, Single-Primer Site-Directed Mutagenesis Method was used (34). We created two types of mutation: stop-removing and start-deletion. For the creation of stop-removing constructions, different types of stop codon mutations were introduced depending on the sequence of the studied gene: substitutions, deletions or insertions of 1/2 nucleotides. In all cases, these mutations lead to the creation of uORFs that overlap with the main CDSs. To create start deletion mutations, we completely deleted the corresponding start codon. The primers used for cloning and mutagenesis are listed in Supplementary Methods Table S3.

### Luciferase assay

For the luciferase activity assay, the wild-type and mutant plasmids were separately transfected into HEK293T using Lipofectamine 3000 (ThermoFisherScientific, USA) according to the manufacturer's protocol. Cells were seeded at 1.5 × 10^4^ cells/well in 96-well poly-l-lysine-coated plates 24 h prior to transfection. We transfected 100 ng of plasmid DNA per well. Forty-eight hours after transfection, *Firefly* and *Renilla* luciferase activity was measured using Dual-Glo^®^ Luciferase Assay System (Promega, USA) in a black 96-well plate. The experiment was performed in at least four independent biological repeats, each containing three technical replicates. Statistical analysis was performed by the two-sided Student's *t*-test using R software (*P*-values were corrected with the Holm–Bonferroni correction method). Data for each gene of interest were presented relative to the wild-type construct as mean ± SEM among biological replicates.

### Implementation of the uORF Annotator to annotate the effect of genetic variants on uORFs

To annotate the effects of genetic variants within uORF sequences, we developed a custom software tool called *uORF Annotator*. The tool takes the following files as input: (i) a user-provided VCF file containing variants to be annotated; (ii) a BED file containing annotated uORFs; and (iii) reference genome FASTA and an annotation in the GTF format with only uORF-containing transcripts. The output of the program consists of an annotated VCF file, as well as plain text (TSV) results table and a BED file containing uORFs affected by truncating or extending variants (see below). During annotation, variants from the input file are overlapped with both uORF intervals and genomic CDS intervals using BEDtools v. 2.26 (35). Then, the effect of the variant on both the uORF itself and the main ORF of the corresponding gene is determined by examining the type of variant (i.e. SNV or indel), its location relative to exon boundaries (i.e. exonic or splice site; deep intronic variants were excluded from the analysis), and the expected change in length or sequence of the uORF product and main gene product. Only variants that fall within the uORF boundaries are reported; optionally, a user may restrict output to variants located outside of the main CDS of the gene.

A BED file containing uORF features affected by variants is constructed only for variants that potentially affect uORF length (nonsense variants, stop loss variants, frameshift indels). To create such a file, a merged feature is constructed from the exonic sequence of the uORF itself, exonic transcript sequence between the uORF stop codon and the start codon of the main ORF, and the CDS of the main gene. Then, the sequence of such merged feature (after introducing the variant) is examined, and the location of the first stop codon is determined. The variant is then classified into one of the four categories depending on the impact on the protein products of uORF and main gene CDS: (i) main_CDS_unaffected - variants that do not change the overlap between uORF and main CDS (ii) overlap_removal—uORF-truncating variants that eliminate the existing overlap between uORF and main CDS (iii) N-terminal_extension—variants that lead to the production of a chimeric protein product of the gene, possessing an extension at the N-terminus resulting from uORF translation; and (iv) out-of-frame_overlap—variants that lead to the appearance of a new overlapping segment between uORF and main gene CDS, with the two sequences translated in different frames. Genomic coordinates of annotated variants and uORFs are given according to the hg38 human genome assembly.

### Machine learning (ML) approaches used for TIS annotation

To benefit from the hand-crafted dataset, we explored two groups of ML models: (i) neural networks (NNs) and (ii) tree ensembles. For the former, we utilized variations of the transformer architecture, such as BERT (36), distilBERT (37) and deBERTa (38). We attempted to use the transfer learning paradigm, where the model is first trained to recover masked input tokens (masked language modeling, MLM) and then fine-tuned on the downstream objective—a TIS prediction in our case. As a second ML paradigm, we used boosted trees of XGBoost (39). The Supplementary Methods provide a more detailed (yet simple) justification for the models’ choices. The data preparation routines differed for these two groups of models and are detailed below.

### Data preparation for ML models

Building the dataset for the model's training and inference required extracting 5'UTR regions of the protein-coding genes. As previously, we utilized CAGE data from the FANTOM5 project (40) to refine transcript start sites, configuring the ORFik package (41) to search for CAGE peaks in the vicinity of 1000 nucleotides around the canonical starts found in the Ensembl annotation. We processed each transcript by removing intronic and concatenating exonic regions. We removed duplicated sequences, thus retaining only unique 5′UTR definitions. We mapped each sequence to genomic coordinates and took a reverse complement for the negative strand to unify direction. 5'UTR sequences varied in length: from 1 to 7910 nucleotides after introns' removal. Thus, we filtered out sequences outside of [30, 3000] boundaries, retaining over 92.6% of the entries.

In total, the dataset encompassed 79453 unique 5'UTR sequences, out of which 19 576 were from manually curated genes and thereby comprised the modeling dataset. We split the latter into the training (80%, 3011 genes, 15947 sequences), validation (10%, 379 genes, 1882 sequences), and testing (10%, 371 genes, 1919 sequences) datasets according to Ensembl gene IDs and accounting for overlapping genes. The number of unique 5′UTRs exceeded the number of genes six-fold: there were 5.27 transcripts with different 5'UTR compositions per gene on average.

As pure sequence-based models resulted in poor performance, we employed additional sequence-level features processed from the experimental data. Namely, we fetched total read counts for each position from ribosomal P-site identification data downloaded from the GWIPS-viz browser website (42). Read counts were capped at 5000 and then linearly scaled between 0 and 10.

The subsequent data preparation routines depended on the ML model used and are exemplified in Supplementary Methods Figure S1. For the NN-based models, we utilized the sliding window approach in two steps: (i) to produce *k*-mers of length three—the subsequent input tokens—and (ii) to split input tokenized sequences into segments of unified size (98 with step 20). We padded tokenized shorter than 98 elements from the right side using a special PAD token. Furthermore, two special tokens, added following the NLP convention, signified each sequence's start (CLS) and end (SEP), resulting in 100-sized inputs. Before training, we filtered out sequence slices without any classes. For the classification objective, we assigned each type of codon present in the curated dataset a binary class following our data. We masked the rest of the tokens nullifying their contribution to the loss.

For the MLM, we utilized two strategies. Due to the overlapping of consecutive k-mers, they had to be masked consecutively. Thus, we randomly picked 5% of the input tokens in the first approach and broadened the selection to two neighboring tokens. As a second approach, to direct the attention mechanism towards putative translation initiation sites, the selection started with these and broadened the same way as above. In both cases, the fraction of masked tokens constituted approximately 15%.

In contrast to the seq2seq nature of the NN-based models, we trained XGBoost on the sentence-level objective. Namely, we centered each input sequence on the start codon and predicted its class. We encoded sequences using the one-hot approach. For instance, a sequence ACGTX is encoded as five vectors: (1, 0, 0, 0), (0,1, 0, 0), (0, 0,1, 0), (0, 0, 0,1) and (0, 0, 0, 0) where the X character is used to denote unknown nucleotides and the padding. Using flank sizes of 50 around the start codon resulted in 101-sized sequences or 404-sized row-vector of the encoded representation. We augmented each sequence with the experimental signal prepared as described above. Finally, for XGBoost, we employed transcript-level features — standardized per-cell expression levels fetched from the human protein atlas (43), reaching 581 total features per sequence.

The sliding window for NNs and centering for the XGBoost increased the modeling dataset size up to 176 456 and 437 970 instances, respectively. During inference and performance assessment, we averaged ML-predicted probabilities based on genomic coordinates and assigned a binary class based on the threshold of 0.5.

To construct the lncRNA inference dataset, we relied on the LNCipedia meta-aggregator database, version 5.2 (44). Namely, we downloaded the exonic lncRNA sequences' genomic coordinates in GFF format, manually extracted the corresponding sequences from the hg38 reference, and concatenated them. Further, we filtered the obtained sequences by the length between 200 and 5000 nucleotides, still encompassing almost all annotated lncRNAs, amounting to a much more extensive quantity of the sequence data than 5'UTRs of the protein-coding genes: 117642 sequences with 626 median size, ∼125M nucleotides in total. The rest of the data preparation routine followed the same steps as above.

### The ML models’ training detailed

Our approach to NN model training followed the mainstream NLP paradigm in using the AdamW optimizer (45) and a linear learning schedule with a short warmup period to prevent early overfitting. Typically, we set the peak learning rate to 10^−4^, the warmup period comprising 1% of the incremental steps across 300 epochs. We used the *transformers* library (30) to obtain the NN models and the *pytorch-lightning* to manage training, clip gradients and mixed precision.

To train and validate the XGBoost model, we used the scikit-learn interface. We concatenated training and validation datasets during cross-validation and generated ten random folds based on Gene IDs.

In both cases, we used loss function weights to handle class imbalance. In the case of NN models, we manually set weights of 1 and 5 for negative and positive (valid TIS) classes based on the observed balance between precision and recall. In the case of XGBoost, the weights (and other key hyperparameters) were optimized with Optuna (46) (refer to Supplementary Methods Table S1 for the resulting parameters and their description).

We used the early stopping technique to avoid overfitting at the later stages of the training process. In the case of NNs, we halted the training if the loss function (cross-entropy) didn’t improve for ten consecutive epochs. In the case of XGBoost, adding decision trees terminated if the last ten added trees did not improve the loss.

## RESULTS

### Manual annotation revealed ∼5.2 thousand of additional translation initiation sites in 1889 OMIM genes

Ribosome profiling technology revealed that many human 5′UTRs could be translated, and there are several lists of potentially translated uORFs (13,14,19). However, these lists have disadvantages that impede their application in identifying potential disease-causing variants in the genetic data of patients with Mendelian disorders. To create a more suitable list of uORFs, we performed a manual annotation of alternative upstream translation initiation sites (TISs) for genes associated with Mendelian disorders.

We took 3641 genes mentioned in the OMIM database: 1242 genes associated with dominant inheritance patterns only and 2399 associated with recessive or both inheritance patterns. We used publicly available data presented in the GWIPS-viz (42) and Trips-Viz (22) browsers to analyze these genes. At first, we checked the level of mRNA expression using the global aggregate of RNA-seq coverage data from 20 different cell types available in the GWIPS-viz. We analyzed transcripts in which the maximum exon coverage was at least 10 times higher than the background coverage of the adjacent intergenic or intronic regions (and was at least 20 in absolute terms). Moreover, we determined the exact 5′-end of mRNA using FANTOM5 CAGE data of the identified transcription start sites (TSSs) (23). The analyzed TSSs were also supported by RNA-seq coverage.

Next, we annotated upstream translation initiation sites (uTISs). For this, we used initiating and elongating ribosomes profiling data from the eight independent studies of the six different cell types and only elongating ribosomes profiling data from additional 39 studies of 23 cell types. These data were presented as tracks in the GWIPS-viz browser and as single transcript plot in the Trips-Viz browser. Additionally, we performed a transcriptome-scale prediction of Kozak-sequence strengths. Since it was previously shown that non-canonical TISs are widespread (47), we analyzed the Kozak consensus sequence both for ATG start codons and for near-cognate codons (NCCs: TTG, GTG, CTG, AAG, AGG, ACG, ATA, ATT, ATC). In addition, we used a list of mouse uORFs created by Wang *et al.* (33) to define conservatively translated uORFs.

Our manual annotation of alternative translation initiation sites was conducted in conformity with the following criteria: (i) a distinct peak of initiating ribosome profiling data; (ii) the identified peak corresponded to distinct elongating ribosomes profiling signal; (iii) the peak matched with Kozak consensus; (iv) Trips-Viz single transcript plot (showing the ORF architecture) confirmed the translation of expected ORF; (v) the presence of a translation of the corresponding uORF in the mouse dataset was strong, but not a mandatory criterion. Algorithm of manual annotation and examples of uTISs detection are shown in Supplementary Figures S3 – S14. Next, we used in-house tool to generate ORFs from annotated TISs based primarily on HGMD RefSeq curated transcripts.

**Figure 1.**
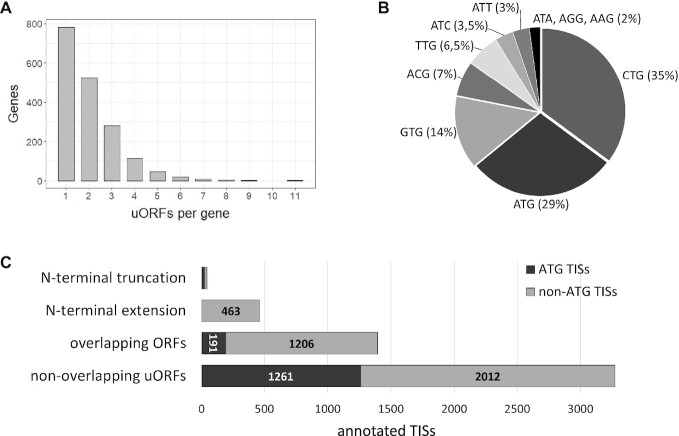
(**A**) Distribution of annotated uORFs in the OMIM genes. (**B**) Start codons for annotated TISs in the 5′UTRs of OMIM genes. (**C**) Annotated TISs are divided into four groups; each group contains both ATG- and non-ATG-started TISs in varying proportions.

As a result, we identified upstream TISs in half of the studied genes (1889), while 20% did not contain additional TISs, and 30% were not covered by available Ribo-seq data. Annotated TISs could lead to the creation of upstream open reading frames, as well as to alternative translation initiation of the gene's main coding sequences. We noted that 22% of uORFs have several TISs but a common stop-codon. According to our annotation, 783 genes contain only one uORF, and 153 out of them have multiple TISs. Additionally, 999 genes contain more than one uORFs (Figure 1A). The maximum uORF number (eleven) was observed in the *NR2F1* gene; in five other genes (*NR2F2*, *CEP250*, *DUSP6*, *EXTL3* and *ABL1*) we identified eight or nine uORFs. Interestingly, all these genes have long 5′UTRs with uORFs evenly distributed from the 5′-end to the reference translation start. This observation hints that such uORFs can regulate the translation of the main gene CDS through cascaded translation reinitiation. Such examples were previously described for other genes, even those containing one or two uORFs (48,49). Besides, we revealed that 389 genes (10,5% of all studied) have alternative TISs for their reference CDS, which leads to both extension and truncation of protein N-termini.

In total, we annotated ∼5.2 thousand additional TISs and divided ORFs they produce into four groups: (i) upstream ORFs that do not overlap with the gene's main coding sequence (non-overlapping uORFs); (ii) upstream ORFs that overlap with the gene's main coding sequence (overlapping uORFs); (iii) alternative TISs leading to extension of reference coding sequence; (iv) alternative TISs leading to truncation of reference coding sequence (Figure 1C, Supplemental data S1, S2). Notably, 71% of all additional upstream TISs corresponded to non-ATG codons (Figure 1B, C). The most commonly used near-cognate codons are CTG (35%) and GTG (14%) (Figure 1B).

### Comparison of manually annotated uORFs with previously published uORFs lists

We compared our manually annotated TISs of uORFs with the previously published studies. We used human uORFs predictions from three studies and considered only uORFs found in the OMIM genes. In total, we included in the comparative analysis: 39 thousand (out of ∼189 thousand) uORFs found by McGillivray *et al.*(14); 1.7 thousand (out of 7.8 thousand) uORFs found by Ji *et al.* (13); and 449 (out of 1933) uORFs found by Scholz *et al.* (19).

We compared ATG-started and non-ATG-started uORFs separately since different studies had different prediction rates for these groups. Only 7.8% of the uORFs predicted by McGillivray *et al.* started with ATG-codon, while Ji *et al.* had 58.6% of them. In our data, 28% of all uORFs were ATG-started. Scholz *et al.* described only ATG-started uORFs.

**Figure 2.**
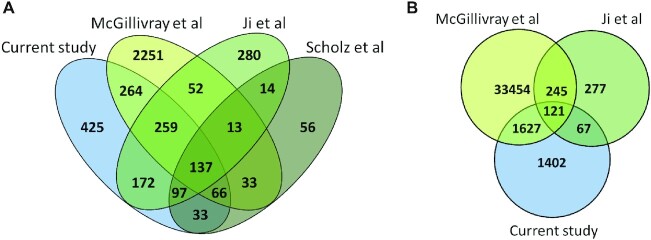
The overlaps of ATG-started (**A**) and non-ATG-started (**B**) uORFs found in the OMIM genes in the current study and McGillivray *et al.* (14), Ji *et al.* (13), Scholz *et al.* (19) studies.

Overall, we analyzed 4152 ATG starts for uORFs from four studies. Comparative analysis revealed that only 137 ATG-started uORFs are present in all four datasets (Figure 2A). 435 ATG-started uORFs were found in three out of four datasets, and 1140 — in at least two datasets. Notably, a major part of ATG-started uORF overlapped between different studies. The only exception is the data obtained by McGillivray *et al.*, where 73% of the uORFs identified by the authors were not part of other datasets. In contrast, 70% of ATG-started uORFs described in the present study were present in other datasets.

Next, we compared the non-ATG-started uORFs from three suitable datasets. In total, the analysis included 37 193 non-ATG-started uORFs. We observed that only 121 uORFs are present in all three datasets (Figure 2B). Any two out of three datasets had 1940 uORFs in common. Thus, we observed that non-ATG-started uORFs overlapped much worse than ATG-started. 94% of the uORFs in the McGillivray's et al. dataset and 44% in our dataset did not intersect with other lists.

Based on the comparative analysis, we created a list of the most reliable uORFs designating them as ‘high confidence uORFs’. In this list we included only uORFs predicted in at least two different studies (Supplemental Data S3). Thus, we identified 1140 ATG-started and 2061 non-ATG-started high confidence uORFs. Interestingly, although the proportion of ATG-started uORFs overlapping between multiple datasets is much higher, the final high-confidence list contains almost two times more non-ATG-started uORFs.

### Analysis of kozak scores and translation initiation level of annotated translation initiation sites

The major group of annotated additional TISs refers to the upstream ORFs. We identified ∼4.7 thousand TISs related to uORFs in 1782 OMIM genes: ∼3.3 thousand for upstream ORFs that do not overlap with downstream CDS and ∼1.4 thousand for upstream ORFs that do. Both uORFs types may significantly affect the translation of the main coding region of a gene by numerous mechanisms, including leaky-scanning, ribosome-stalling, and translation reinitiation (2). However, the effect of the overlapping uORFs seems to be more pronounced since they directly compete for ribosome binding with downstream CDSs. Thus, we assumed that the features of TISs belonging to distinct uORF groups should differ.

**Figure 3.**
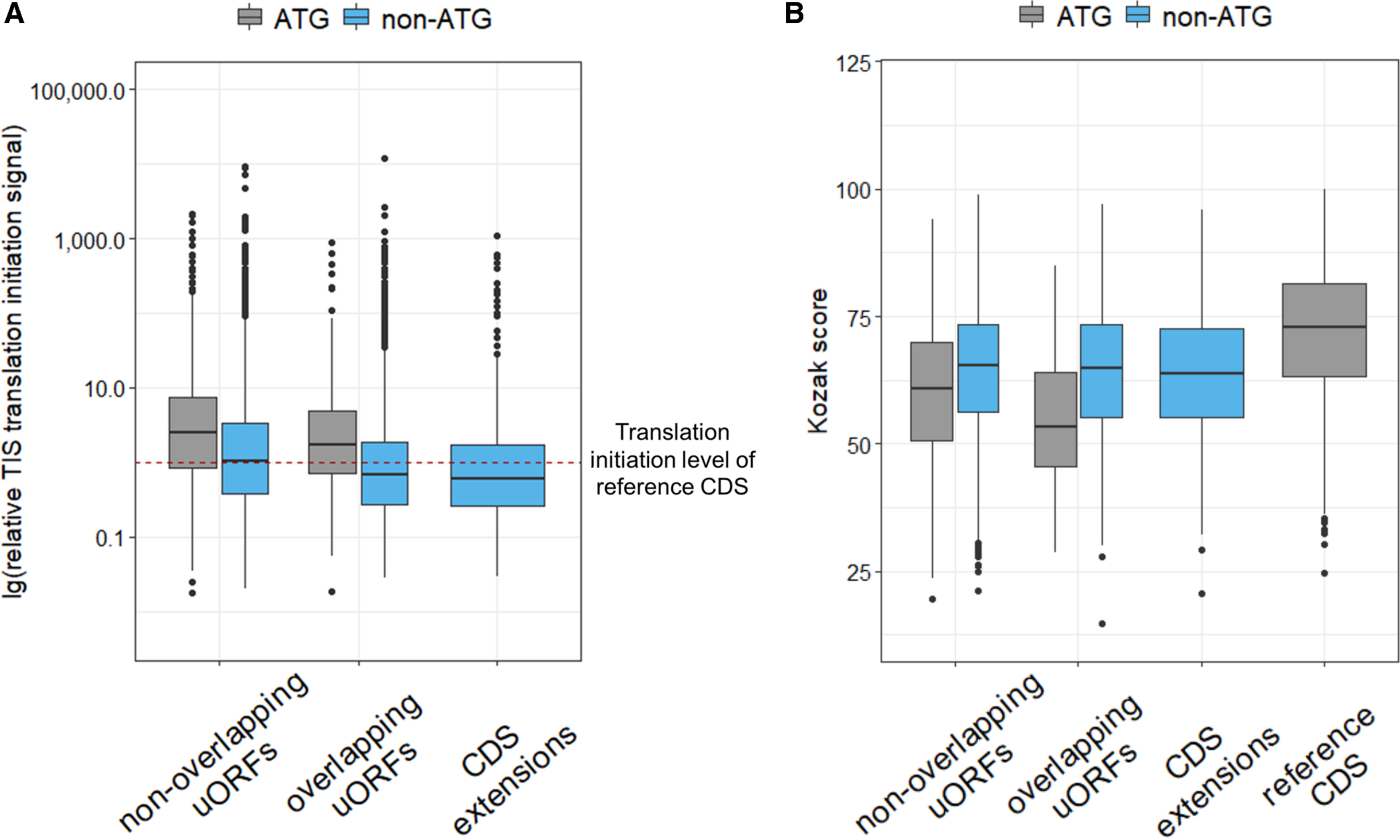
Comparison of relative translation initiation signal according to initiating ribosome profiling data from the GWIPS-viz browser (**A**) and comparison of Kozak score calculated without taking into account the start codon (**B**) between different groups of uORFs and N-terminal CDS extensions.

We revealed 38% of the canonical ATG TISs among the non-overlapping uORFs, while there were only 13.5% among overlapping uORFs (Figure 1С). We analyzed the translation initiation signal of each uORF start relative to the corresponding start of downstream CDS, using initiating ribosome profiling data from the GWIPS-viz browser. This analysis revealed that half of all uORF TISs had the translation initiation signal equal to or higher than the main protein-coding ORF. Moreover, we found that overlapping uORFs had lower relative translation initiation levels compared to non-overlapping (*P*-value = 6.41x10^−23^, Wilcoxon two-sided Rank Sum test), while non-ATG TISs had lower relative translation initiation levels than ATG-starting in both uORFs groups (non-overlapping: *P*-value = 6.49x10^−22^; overlapping: *P*-value = 1.76x10^−9^, Wilcoxon two-sided rank sum test) (Figure 3A).

We have also analyzed the Kozak context for different uORFs TISs. We used the Kozak score calculated without taking into account the start codon. It means that our Kozak score reflects only the surrounding context of the analyzed start codon. We compared Kozak scores of different uORFs groups and reference gene CDSs (Figure 3B). Notably, all groups of uORFs had a significantly lower Kozak score than reference CDSs (*P*-value = 1.46x10^−147^, Wilcoxon two-sided rank sum test). Considering only ATG-started uORFs, we observed a stronger decrease in the Kozak score. This data may suggest a negative selection against strong TISs in 5′UTRs. However, we demonstrated that the uORFs translation initiation level is often higher compared to corresponding CDSs (Figure 3A). For example, 69% of ATG-starting uORFs had translation initiation levels higher than the downstream CDS, while only 38% of them had a higher Kozak score. This observation is consistent with the fact that during scanning, the ribosomal preinitiation complex (PIC) preferably recognizes the first suitable start codon rather than the start codon in a stronger Kozak context.

Next, we compared Kozak scores between different uORFs groups (Figure 3B). We did not observe a difference in Kozak context score between overlapping and non-overlapping non-ATG-started uORFs (*P*-value = 0.7961, Wilcoxon two-sided rank sum test) while overlapping ATG-started uORFs had lower Kozak score than non-overlapping (*P*-value = 3.11x10^−05^, Wilcoxon two-sided rank sum test). Further analysis of Kozak scores revealed that ATG-started uORFs had significantly lower Kozak scores than non-ATG-started (*P*-value = 1.43x10^−28^, Wilcoxon two-sided rank sum test) (Figure 3B), in contrast non-ATG uORFs had lower translation initiation level than ATG-started (Figure 3A). This observation confirms that the presence of an optimal Kozak context is important for the efficient use of near-cognate start codons, what was shown in previous studies (50).

In addition, we identified 506 alternative TISs related to the main CDSs in 386 out of the 3641 OMIM genes. 463 alternative TISs lead to the extension of a reference coding sequence. As expected, there was no CDS extension started with ATG-codon since the standard annotation process focused on the longest gene ORFs. Analysis of the relative translation initiation signal obtained from initiating ribosome profiling data revealed that most of the CDS extensions had lower translation initiation signals than corresponding reference starts (Figure 3A). The Kozak sequence strength of the extended TISs was also significantly less than the reference (*P*-value = 1.29x10^−31^, Wilcoxon two-sided rank sum test) and did not differ from the TISs of non-ATG starting uORFs (Figure 3B).

Other 43 annotated TISs lead to the main CDS truncation, and half of these truncations (51%) are ATG-related. Interestingly, two-thirds of the CDS truncations are related to mRNA 5′-end isoforms. In such genes, at least one of the transcription start sites is located downstream from the reference translation start, according to FANTOM5 CAGE data. Thus, these start sites are not true alternative translation initiation start sites. Most of the other CDS truncations are TISs located very close to the reference start and having a comparable translation initiation signal, and such starts should not lead to a significant change in the protein structure.

### Experimental validation of predicted uORFs translation

For experimental validation, we selected eight predicted uORFs: two overlapping and six non-overlapping uORFs. We cloned the full-length 5′UTRs with the few first codons of the studied genes into the psiCHECK-2 vector. In obtaining wild-type plasmids, the 5′UTR was fused with *Renilla* luciferase so that the CDS of luciferase started with the reference start codon of the gene. Simultaneously, the original vector-derived luciferase start codon was eliminated from the final plasmids. Next, we introduced uORF-disrupting mutations in each construct and analyzed their effect on the translation of the reference CDS using a dual luciferase assay in the HEK293T cell line.

**Figure 4.**
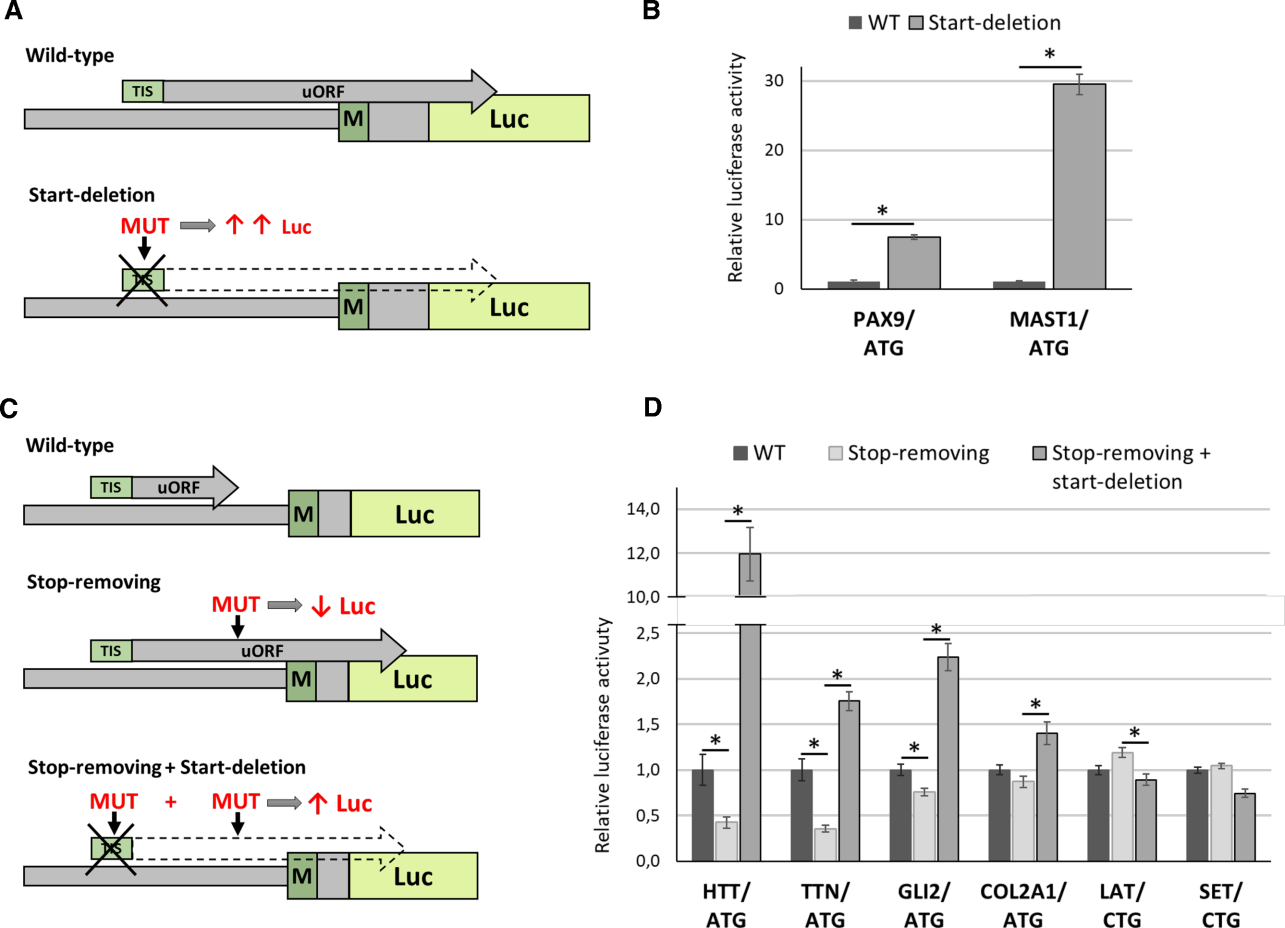
Experimental validation of translation for annotated overlapping (**A**, **B**) and non-overlapping (**C**, **D**) uORFs. (A, C) The schemes of the plasmids used for the experiment. (B, D) Results of the dual-luciferase reporter assay in HEK293T cells transfected with each plasmids for the indicated genes. The start-codons of the analyzed uORFs are listed below the gene name. Data are represented relative to the WT construct as mean ± SEM (*P < 0.05).

To analyze overlapping uORFs, we deleted their start codons. Such ‘start-deletion’ mutations prevent the translation of overlapping uORFs and, therefore, should lead to an increase in luciferase level (Figure 4A). We analyzed two ATG-started overlapping uORFs in the *PAX9* and *MAST1* genes. *MAST1* uORF was predicted exclusively in the present study. *PAX9* uORF was revealed in the present and Jin *et al.* (13) studies. We performed luciferase experiments and observed a strong increase in the luciferase level for both overlapping uORFs (Figure 4B). Thus, we experimentally confirmed the translation of ATG-started overlapping uORFs in the *PAX9* and *MAST1* genes. Pathogenic variants in these genes lead to Tooth agenesis (*PAX9*) and Mega-corpus-callosum syndrome with cerebellar hypoplasia and cortical malformations (*MAST1*). In the present study, we showed that disruption of overlapping uORFs in these genes could significantly affect protein expression. However, it remains unclear whether the nucleotide variants in these overlapping uORFs can lead to Mendelian disorders. This phenomenon is of interest for further investigation.

Another group of uORFs is uORFs that do not overlap with the downstream gene CDSs. For experimental analysis of non-overlapping uORFs, we first disrupted their stop codons. Such ‘stop-removing’ mutations lead to a formation of extended uORFs that out-of-frame overlap with the downstream CDSs and, therefore, should lead to a decrease in luciferase level. Next, we deleted the start-codons of the resulting overlapping uORFs, which should increase the luciferase translation (Figure 4C). We analyzed four ATG-started uORFs in the *HTT, TTN, GLI2* and *COL2A1* genes and two CTG-started in the *LAT* and *SET* genes. For the *HTT, TTN* and *GLI2* genes, we observed strong expected effects for both stop-removing and start-deletion mutations (Figure 4D) and therefore validated the existence of ATG-started uORFs in these genes. For another ATG-started uORF in *COL2A1*, we did not observe a statistically significant effect for stop removal mutation. However, the subsequent deletion of the start-codon resulted in a significant 1.5-fold increase in luciferase translation level (Figure 4D). This led us to assume that uORF in the *COL2A1* gene exists, but its effect on downstream CDS translation level is low, at least in our model system.

Thus, we experimentally confirmed the translation of ATG-started uORFs in the *HTT*, *TTN*, *GLI2* and *COL2A1* genes. Three of them were predicted exclusively in the present study (in *TTN*, *GLI2* and *COL2A1)*. Moreover, we showed that mutations disrupting uORFs in the *HTT*, *TTN* and *GLI2* could lead to a decrease in the main protein expression, resulting in a loss or reduction of gene function. Pathogenic variants in these genes cause Huntington's disease (*HTT*), some cardiomyopathies and muscular dystrophies (*TTN*), Culler-Jones syndrome, and Holoprosencephaly 9 (*GLI2*). It was previously shown that loss-of-function variants in the *GLI2* and *TTN* genes lead to the development of the disease (51,52). Thus, we suggested that nucleotide variants that disrupt uORFs in these two genes could potentially cause Mendelian disorders.

For non-ATG-started uORFs, the expected effects of mutations on luciferase translation were not detected at all (Figure 4D). Although some mutations resulted in slight changes in luciferase activity, the direction of these changes did not match those mediated by uORF, suggesting that these changes are not related to uORFs. We noted that *LAT* uORF was presented exclusively in our uORF dataset, while *SET* uORF was also identified in the previous study and, therefore, classified as ‘high confidence.’ However, we did not detect the translation of both of these uORFs. We hypothesized that the lack of effect for non-ATG uORFs in our experiment may be associated with a pronounced tissue specificity of their expression or the presence of other regulatory elements that can affect the translation of the main CDS. Previously, Wang *et al.* showed that uORF expression is highly tissue- and developmental stage-specific in mice (33). Moreover, analysis of initiating ribosome profiles for individual experiments from GWIPS-Viz and Trips-Viz browsers revealed that *LAT* and *SET* uORFs’ TISs are indeed present only in some cell types. Another reason for the lack of the effect for non-ATG uORFs may be that such uORFs are translated much less efficiently compared to ATG-started main gene CDS. Therefore, the translation level of the created overlapping uORF may not be sufficient to significantly suppress the translation of the main CDS.

### Experimental validation of predicted N-terminal extensions of CDS in the OMIM genes

**Figure 5.**
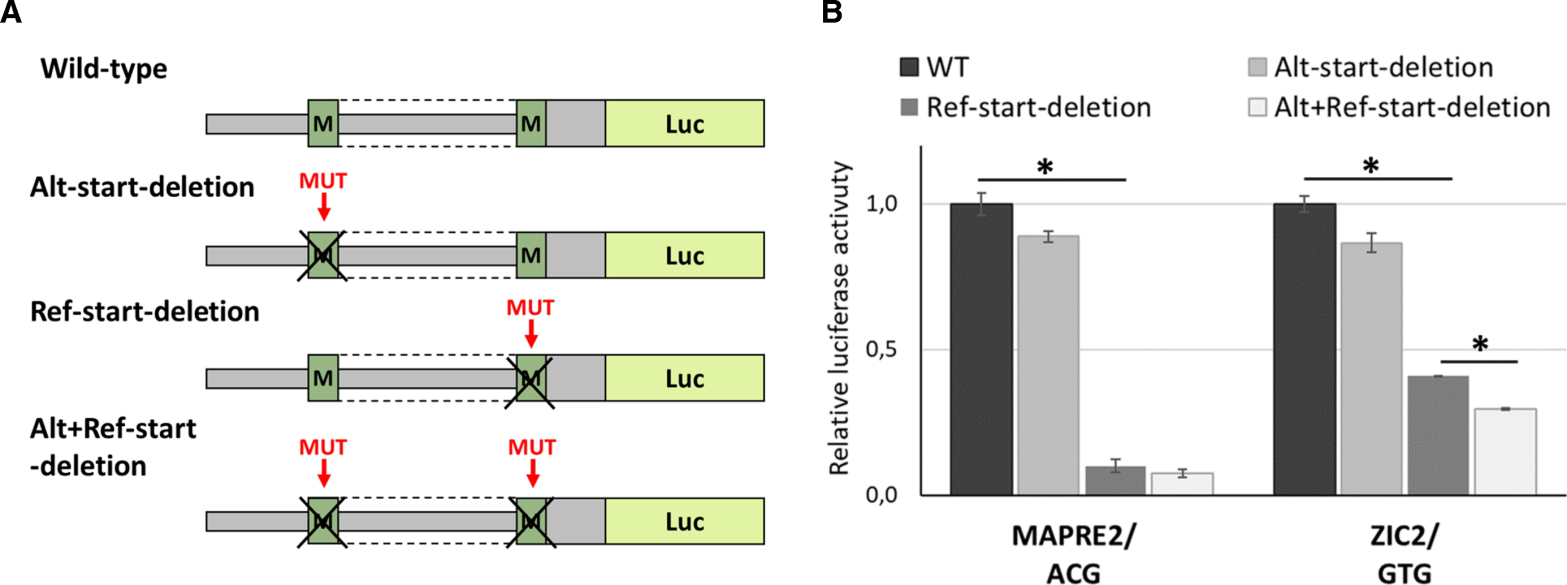
Experimental validation of annotated non-ATG-started N-terminal extensions of the *MAPRE2* and *ZIC2* CDSs. (**A**) The schemes of the plasmids used for the experiment. (**B**) Results of the dual-luciferase reporter assay in HEK293T cells transfected with each plasmids for the indicated genes. The start-codons of the analyzed uORFs are listed below the gene name. Data are represented relative to the WT construct as mean ± SEM (*P < 0.05).

All annotated TISs associated with N-terminal CDS extensions started with non-ATG codons. We selected two such extensions in the *MAPRE2* and *ZIC2* genes for experimental validation. For this, we created plasmids containing wild type 5′UTR of studied genes fused with *Renilla* luciferase as described above. Next, we created three types of mutated plasmids: with the deletion of the alternative predicted start-codon, with the deletion of reference start-codon, and with the deletion of both start-codons. Thus, we assessed the contribution of both TISs to the total protein translation level using dual luciferase assay in the HEK293T cell line (Figure 5A).

Experimental analysis of *MAPRE2* revealed an insignificant decrease in the luciferase activity for deletion of alternative TIS, while the removal of reference start-codon resulted in a 10-fold decrease of luciferase level. (Figure 5B). This suggests that our experimental system cannot reliably detect *MAPRE2* N-terminal extension. Analysis of *ZIC2* revealed a slight (11–13%) but significant decrease in the luciferase activity upon deletion of alternative TIS, at least in the context of the absence of the reference start codon. However, the effect was much weaker compared to the deletion of reference ATG, leading us to conclude that the translation of a small fraction of the ZIC2 protein started with an alternative GTG-start codon, at least in our model system.

### Annotation of genetic variants affecting uORFs

**Figure 6.**
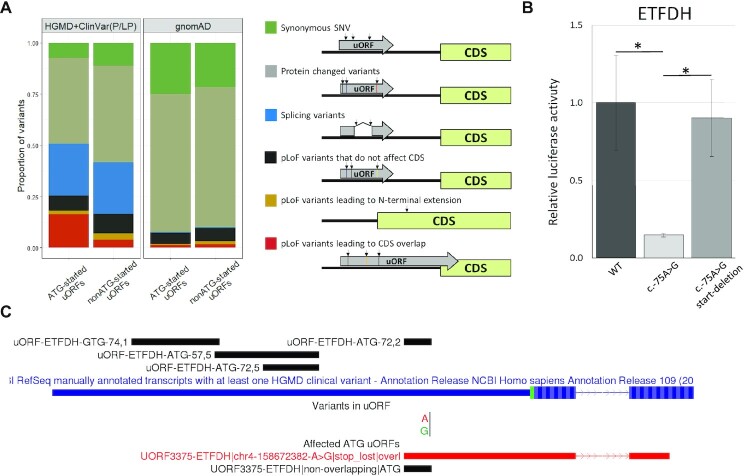
Analysis of pathogenic and likely pathogenic variants from the HGMD (DM) and ClinVar (P/LP) databases, as well as population variants from the gnomAD database, using uORF Annotator. (**A**) The proportion of different types of genetic variants affecting ATG- and non-ATG-started uORFs among pathogenic/likely pathogenic variants (HGMD + ClinVar) and population variants from the gnomAD. The consequences of different types of variants are schematically depicted to the right of the diagram. (**B**) The scheme of annotated uORFs in the *ETFDH* gene (upper) and visualization of uORF Annotator prediction results (below). Track ‘Variants in uORFs’ displays the annotated variant; track ‘Affected ATG uORFs’ shows the original uORF in which the variant is localized (black) and the resulting uORF (red). (**C**) Experimental analysis of c.-75A > G variant in the *ETFDH* uORF using dual-luciferase reporter assay in HEK293T cells. Data are represented relative to the WT construct as mean ± SEM (*P < 0.05).

Several previous studies revealed genetic variants that disrupt the existing uORFs and thereby lead to Mendelian disorders and malignancies (8,11). In the present study, we implemented a software tool to annotate the effects of genetic variants located in annotated uORFs. It takes a VCF file with the variants of interest as input. In addition to the annotated VCF file, the tool generates a BED files that can be loaded into the genomic browser (e.g. GWIPS-viz, UCSC, IGV) to visualize the effect of a variant on the uORF structure (Figure 6B). We used our tool to evaluate the effects of known pathogenic variants from HGMD Pro 2021.3 (24) and ClinVar database (from 2022-01-29) (25) as well as variants present in the Genome Aggregation Database (gnomAD) v3.1.2 genomes (26).

Excluding deep intronic variants and large deletions/insertions, 123 pathogenic (disease-causing - DM) variants from HGMD and 83 pathogenic or likely pathogenic variants from ClinVar were found to be located in the 5′UTRs and affect uORFs (Supplemental Data S4). Of these, the most prevalent class was missense variants (53 in HGMD and 34 in ClinVar). Together with other variants altering the amino acid sequence of the uORFs (i.e. in-frame deletions/insertions, stop-gained), they represented 47% and 51% of the variants from HGMD and ClinVar, respectively (Figure 6A).

The most commonly described pathogenic variants that affect uORFs are frameshift and stop loss variants (9,11). Such variants lead to the formation of extended uORFs that may overlap with the downstream protein-coding sequences and thereby reduce their translation. We classified these variants as probably leading to loss of gene function (pLoF) and additionally analyzed how the resulting change in the uORF structure affects the main coding part of the gene. For this, frameshift and stop loss variants in the uORFs were divided into three groups: (i) variants leading to translation of uORF that do not affect reference CDS; (ii) variants leading to N-terminal extension of CDS; (iii) variants producing uORF that out-of-frame overlaps with the downstream CDS (Figure 6A). We observed 16 pLoF pathogenic variants in HGMD and 13 in ClinVar, of them 11 and 4, respectively, resulting in the creation of overlapping uORFs. Some of them were previously described in the literature in relation to uORF disruption, e.g. in the *PAX6* gene (11). Besides, we identified an uncharacterized stop loss variant (chr8:22130605T>C) located in the previously described uORF in the *HR* gene (10). We also revealed pLoF variants in previously undescribed uORFs, e.g. stop loss variant (chr4:158672382A >G) in the *ETFDH* uORF (Figure 6B) and frameshift variant (chr22:29603933A >AT) in the *NF2* uORF. Both of these variants result in the formation of extended ATG-started uORFs that overlap with the downstream CDSs. Thus, we suggest that the pathogenicity of these variants is associated with the disruption of uORFs.

To confirm this hypothesis for the *ETFDH* gene, we conducted luciferase experiments for the chr4:158672382A >G (NM_004453.4:c.-75A >G) variant. This variant was described as the cause of the Multiple acyl-coenzyme A dehydrogenation deficiency (MADD) (MIM # 231680) (53). The experiments were performed according to the scheme described above (Figure 4C). Instead of the model stop-removing variant, we introduced a real variant identified in a patient (c.-75A >G). Using luciferase assay in HEK293T cells, we showed that the studied variant led to a significant reduction of the main CDS translation (Figure 6C). Further removal of the start-codon in the context of the variant led to a rescue of the protein translation level (Figure 6C). Thus, we showed that the c.-75A >G variant in the *ETFDH* gene leads to a decrease in the expression of the main protein through the disruption of uORF.

Another intriguing class of uORF-affecting variants is splice variants inside the 5′UTR introns. These variants also can lead to uORF frameshift (11); however, their effect on other mechanisms of gene expression regulation should be considered.

We also used our uORF Annotator to evaluate the effects of variants of uncertain significance (VUS) from ClinVar. 1881 variants were found to affect the uORF sequences, excluding deep intronic variants and large deletions/insertions (Supplemental Data S5). Among them, we identified 101 variants belonging to the uORF pLoF class (42 in ATG-started uORFs and 59 in non-ATG-started uORFs). 22 of them lead to the formation of overlapping uORFs (8 in ATG-started uORFs) and 18 lead to N-terminal extensions (7 in ATG-started uORFs). We hypothesize that many of these variants could be reclassified as likely pathogenic after detailed analysis and experimental validation.

Next, we used our tool to analyze gnomAD variants from a healthy population. We identified 75 319 variants affecting uORFs. Of these, 68% were protein-changed variants, and 22% were synonymous (Figure 6A). The differences in the proportions of the main variant classes (three types of pLoF variants, protein changed variants, splice site variants, and synonymous variants) between gnomAD and pathogenic HGMD/ClinVar were highly significant (*P* ≪ 0.001 in Fisher's exact test) (Figure 6A). uORF pLoF and splice variants were more frequent among pathogenic variants. Although splicing variants can realize their pathogenicity through different mechanisms, the pathogenicity of pLoF variants is most likely associated with the uORFs.

In addition, we compared the proportions of different variants between ATG and non-ATG-started uORFs (Figure 6A). We found that, in the case of pathogenic variants, pLoF variants were less frequent in non-ATG-started uORFs compared to ATG-started uORFs, while vice versa in case of gnomAD. Moreover, this difference is enhanced when considering the most ‘high-impact’ group - variants that lead to CDS overlap (Figure 6A). In the previous sections, we showed that there are more non-ATG uORFs. However, they overlapped much worse between different studies (Figure 2B) and generally had a lower relative level of translation initiation (Figure 3A). Besides, in our experiments, we did not detect non-ATG uORFs translation, as well as their effect on downstream CDSs, probably due to their tissue specificity or low translation level (Figure 4D). To sum up, we hypothesized that variants in the non-ATG-started uORFs may be less significant in the context of Mendelian disorders.

### Models' training for TIS prediction

We used our manually curated data to perform translation initiation sites (TIS) prediction in other genes. In attempts to create a TIS prediction model relying solely on sequence data, we extensively experimented with various NN architectures. We explored two principal approaches: (i) token-level prediction, where the model outputs probabilities for each sequence token (*k*-mer of size three) and (ii) sequence level, where input sequences are centered on valid start codons, and the model learns to infer whether they constitute valid TIS. Note that all transformer models in this study require a single pretrained model to leverage both tasks. As a result, we trained the following architectures: BERT (36), DeBERTa (38), and distilBERT (37) on sequence for both of these tasks (and FunnelTransformer (54) for the sentence-level classification only): using and omitting the pretraining phase on the MLM objective. Furthermore, we attempted to benefit from the DNABERT model pretrained on DNA sequences using the exact size (3) of the *k*-mers. To our surprise, none of these ventures achieved acceptable performance, reaching a maximum of 0.25 *F*1 score.

Hence, we employed Ribo-seq-derived experimental data that served as one of the critical criteria during manual curation. Using it, we focused on token-level prediction with the distilBERT model and sentence-level classification using XGBoost. Further experimentation with the distilBERT revealed that (i) pretraining on the MLM task didn’t yield substantial benefit while drastically increasing the model's complexity and (ii) lowering this complexity by reducing both the size of the internal representations and the number of layers resulted in a better generalization. The resulting distilBERT model (Supplementary Methods Figure S2) had 96K trainable parameters, which was minuscule compared to over 40M of the default architecture.

Following the hypothesis that a simpler model may be more suitable for our purpose, we trained the XGBoost algorithm on a sentence-level classification objective. Due to affordable computational complexity, we employed hyperparameters’ optimization targeted at the best average *F*1 score and cross-validated its performance (Supplementary Methods Table S2).

Table 1 provides a comparison of our predictions to the aforementioned datasets published by Ji *et al.* (13), McGillivray *et al.* (14), and Scholz *et al.* (19) on our test dataset using overlapping start codons in terms of F1 score, precision (PRC), recall (REC) and balanced accuracy (BAC) (Supplementary Methods provides definitions and intuitions for performance metrics). Firstly, XGBoost outperformed distilBERT using only 225 trees and provided a classifier with balanced precision and recall. Overall, the performance correlated with the number of positive examples, except for the ATC start codons. For well-represented TISs, the *F*1 score fluctuated between 0.6 and 0.7. XGBoost resulted in better precision, while distilBERT yielded higher recall.

**Table 1.** Models’ performance and comparison to published data on the test dataset

	*F*_1_	PRC	REC	BAC	TN	FN	FP	TP
			XGBoost					
CTG	0.73	0.67	0.79	0.89	5600	40	75	154
ATG	0.76	0.70	0.82	0.89	1887	31	59	139
GTG	0.66	0.61	0.72	0.85	3759	24	39	61
ACG	0.69	0.65	0.73	0.86	1400	12	17	32
TTG	0.66	0.62	0.70	0.85	3042	13	19	31
ATC	0.80	0.80	0.80	0.90	1918	5	5	20
ATT	0.55	0.44	0.73	0.86	2347	4	14	11
ATA	0.55	0.43	0.75	0.87	1307	1	4	3
AAG	0.00	0.00	0.00	0.50	3762	2	1	0
AGG	0.00	0.00	0.00	0.50	5015	1	2	0
			distilBERT					
CTG	0.64	0.50	0.88	0.93	5502	23	173	171
ATG	0.62	0.48	0.88	0.90	1783	20	163	150
GTG	0.63	0.51	0.81	0.90	3733	16	65	69
ACG	0.60	0.47	0.82	0.89	1376	8	41	36
TTG	0.58	0.51	0.68	0.84	3032	14	29	30
ATC	0.68	0.57	0.84	0.92	1907	4	16	21
ATT	0.37	0.26	0.60	0.79	2336	6	25	9
ATA	0.00	0.00	0.00	0.50	1307	4	4	0
AAG	0.00	0.00	0.00	0.50	3758	2	5	0
AGG	0.00	0.00	0.00	0.50	5012	1	5	0
			McGillivray *et al.*					
CTG	0.17	0.10	0.51	0.68	4839	96	836	98
ATG	0.37	0.33	0.43	0.68	1796	97	150	73
GTG	0.12	0.07	0.51	0.68	3225	42	573	43
ACG	0.14	0.08	0.55	0.68	1150	20	267	24
TTG	0.11	0.06	0.57	0.72	2654	19	407	25
ATC	0.10	0.05	0.48	0.69	1713	13	210	12
ATT	0.05	0.03	0.47	0.68	2113	8	248	7
ATA	0.04	0.02	0.50	0.71	1206	2	105	2
			Ji *et al.*					
CTG	0.08	0.29	0.05	0.52	5653	185	22	9
ATG	0.45	0.78	0.32	0.65	1931	116	15	54
GTG	0.12	0.35	0.07	0.53	3787	79	11	6
TTG	0.07	0.15	0.05	0.52	3050	42	11	2
ATC	0.00	0.00	0.00	0.50	1923	25	0	0
			Scholz *et al.*					
ATG	0.29	0.77	0.18	0.59	1937	140	9	30

For comparison with other authors, we treated our hand-crafted dataset as ‘ground truth’. This and us formulating the learning objective as identifying every potential TIS, regardless of whether it resides in a valid open reading frame (that is, ignoring stop-codons), which differs from the classical uORF definition used by other authors, may have impacted drawn comparisons. Predictions were highly skewed towards false positives for McGillivray et al. and false negatives for Ji *et al.* and Scholz *et al.*

We utilized our XGBoost model on the inference dataset comprising 5'UTR sequences from the 15 066 genes that did not undergo our manual curation process. We estimated 23 985 out of 110 0276 TIS as functional. These potentially active TIS are scattered across 14 772 unique 5'UTR exonic transcript sequences of 7836 genes.

We also performed TIS prediction for human lncRNA. Interestingly, the lncRNA inference dataset rendered a much lower positivity rate: applying the same threshold, our model labeled 9746 (0.14%, in 4689 genes) of nearly 7M putative sites as positive. Some of the genes (e.g. lnc-ATP6V1G2-DDX39B-1:2, lnc-PITX1-4:2, lnc-OR4F16-8:1) had up to twenty predicted TISs, comprising intriguing cases for further investigation. Furthermore, while the most popular CTG comprised the majority of predicted TIS among the 5'UTR sequences, this balance shifted towards the canonical ATG start codon for lncRNA.

## DISCUSSION

To date, the efficiency of genetic diagnosis of Mendelian disorders does not exceed 50%, partly because only coding exons and splicing regions are sequenced and analyzed during routine procedures. Whole-genome sequencing can detect almost any genetic variant; however, the interpretation of their pathogenicity remains a critical challenge. Thus, accurate annotation of the regulatory elements in the human genome is essential for the diagnosis of hereditary diseases.

The 5'UTRs of the genes contain elements important for the translation regulation of a transcript. Upstream open reading frames (uORFs) are one of those elements. It was previously shown that pathogenic variants disrupting the uORFs could lead to various diseases (8,9,11). However, all these studies are devoted to in-depth detailed analysis of a particular gene, and there is still no universal algorithm for the analysis and interpretation of 5'UTR variants. There have been several previous studies that described a large number of uORFs in human genes (13,14,19). However, uORFs from different studies do not overlap well since they were obtained based on different Ribo-seq datasets, while uORFs expression is highly tissue- and developmental stage-specific (33). Moreover, due to the complexity and heterogeneity of translational regulation and the absence of reliable standards for testing computational approaches, manual annotation has the potential to significantly improve data quality compared to classical bioinformatics analysis.

In the present study, we attempted to obtain high-quality data by manually annotating upstream translation initiation sites (uTISs) in the OMIM genes. For this, we analyzed a large amount of high-quality Ribo-Seq data presented in user-friendly GWIPS-viz and Trips-Viz web browsers (21,22). One of the main criteria for annotating TISs was the presence of a distinct peak of initiating ribosome profiling data. These data were obtained during ribosome profiling of cells treated with lactimomycin (LTM) that preferentially acts on the 80S initiation complex when its E-site is empty. Although initiating ribosome profiling allows enrichment for the 80S ribosome at the start codon, this procedure can cause a number of artifacts (17). Therefore, we assessed the ribosome profiling signal corresponding to the translation initiation peak and verified the translation of expected ORF using a Trips-Viz single transcript plot.

Another strict criterion was the matching of the initiating peak with a potential start codon and Kozak context. We calculated Kozak score of surrounding nucleotides equally for ATG and non-ATG start-codons. Although previous study indicated that the optimal context for different start-codons is not exactly the same (55), these differences were not crucial or contradictory. Thus, our Kozak strength prediction is suitable for annotating TISs and subsequent analysis, even though it is not perfect.

We annotated ∼5.2 thousand additional TISs, ∼4.7 thousand of which were related to uORFs (Supplemental Data S1, S2). Based on initiating ribosome profiling data, we revealed that half of uORF TISs had a translation initiation signal equal to or higher than the main protein-coding ORF. Moreover, we found that this signal depends on uORF type and start-codon. Although the peaks of initiating ribosome profiling are not a perfect measure of the translation initiation and elongation efficiency, our observations suggest that uORFs are actively translated in a large number of genes.

In addition, we created a list of ‘high confidence’ uORFs based on a comparative analysis of different studies (Supplemental Data S3). To the best of our knowledge, this constituted the first attempt to obtain ‘clean’ data that can be used in interpreting the variants’ pathogenicity.

Next, we implemented a software tool (*uORF Annotator*, available at https://doi.org/10.5281/zenodo.7435228 and https://github.com/bioinf/uORF_annotator/) to evaluate the effects of genetic variants located in manually annotated uORFs. We identified more than 150 pathogenic genetic variants that affect the uORFs. Dozens of them disrupted uORFs’ structure. We also found variants of uncertain significance that disrupt uORFs and require detailed functional analysis for their possible reclassification. Our experiments, as well as previous studies (9,11), showed that such variants can significantly affect main CDS translation. This is also consistent with Whiffin *et al.* study in which the authors showed that variants disrupting uORF stop codons are under strong negative selection (12). Thus, it is important to consider both the location of the uORF relative to the CDS and the type of uORF variant. All these features determine the activation of different mechanisms that can affect the translation of the main protein-coding region, which can lead not only to the suppression of expression but also to its enhancement. For example, variants that disrupt non-overlapping uORFs and result in reduced translation of the main protein of certain genes can lead to the development of Mendelian diseases through a haploinsufficiency mechanism. On the other hand, variants that disrupt overlapping uORFs and lead to upregulation of protein expression of oncogenes may play a role in the cancer development.

Recently, UTRannotator tool was described for annotating variants in the 5′UTRs (56). A distinctive feature of our uORF Annotator is the visualization of the influence of annotated variants on the uORFs structure. For this, our tool generates BED files (separately for ATG and non-ATG-started uORFs) that can be loaded into the genomic browser (e.g. GWIPS-viz, UCSC, IGV). Such visualization facilitates the analysis of the tool results, the formulation of the hypotheses about the pathogenicity mechanism of the analyzed variant, and design of the experiments.

According to our bioinformatic and experimental analysis, variants in the ATG-started uORFs have a more pronounced effect on CDS translation compared to non-ATG started. However, during annotation, we found more non-ATG uORFs, even considering only ‘high confidence’ uORFs. Moreover, many previous studies confirm this trend. For instance, Na *et al.* observed many non-ATG alternative translation initiation sites (47). The authors showed that the most commonly used non-ATG start codons were CTG and GTG, which also coincides with our data. In a study provided by McGillivray *et al.*, the CTG start was also the most frequently used (14). Moreover, it was experimentally shown that the decrease in the translation activation signal depends on the start codon in the following order: AUG > CTG > GTG > TTG (50) although the order of non-ATG TIS activity may vary depending on the nucleotide context (55). The fact that we and other authors found many non-ATG uORFs demonstrates the great interest in their further study. However, in the context of Mendelian disorders, their role may be less significant compared to ATG-started uORFs. Firstly, because non-ATG uORFs are translated much less efficiently compared to ATG-started, and therefore their effect on the translation of the main ATG-started CDS may not be strong enough to cause Mendelian disorder (55). We also assumed that due to this fact, we did not observe the expected effect of ‘stop-removing’ mutations in our experimental system for non-ATG uORFs. Another possible reason for this is that the non-ATG Kozak sequences have tissue-specific translation initiation features and should be studied in suitable biological models.

Analysis of the 5'UTRs regions also revealed TISs related to changes in the reference CDS length, which are of particular interest. In many cases, this was due to incorrect annotation of the mRNA structure of RefSeq genes. Quite often, we observed CAGE peaks indicating the transcription start site, either far upstream of the annotated 5'-end of the mRNA or vice versa within the mRNA sequence. However, we found more than 400 cases of a possible extension of CDS. We showed that translation of protein-coding ORFs can begin with a non-ATG-started Kozak sequence. Our experiments partially confirmed this, since it is likely that non-ATG TISs have low translation initiation efficiency or are expressed in a tissue-specific manner. We found far fewer potential CDS truncations than extensions, and most of them are ATG-started. In many cases, these truncations are quite small in size and affect a few amino acids. This is consistent with previous observations that ATG triplets occur more often in the same frame downstream of weak CDS starts than of strong starts. This may help reduce synthesis of wasteful out-of-frame product and also aid the production of multiple proteoforms from certain mRNAs (57). Thus, our observations demonstrate that correct annotation of the coding part of the OMIM genes is crucial since it is directly related to the interpretation of the variants found in patients with hereditary diseases. Therefore, an in-depth investigation of such cases is important for medical genetics.

Due to the limited number of tissues with available Ribo-seq data, we were unable to analyze all OMIM genes. Moreover, due to the high labor intensity of manual TIS annotation, we did not analyze most of human genes. Therefore, we decided to develop a machine-learning algorithm that allows us to predict the uORF for all human genes. We used our uORFs dataset to train a neural network discriminating between translation start sites. The model was trained on unique 5'UTR DNA sequences, each providing a unique context for the putative TIS, and internally used the experimental Ribo-seq signal.

Our attempts to create a pure sequence-based model have been unsuccessful so far. We acknowledge that having such a model would immediately broaden the applicability domain to allow, e.g. deep mutational scanning of 5'UTR sequences of various organisms. Hence, we suspect our current predictions contain substantial false negatives in the regions less covered by the experimental data. On the other hand, we are more confident in our positive predictions since the RiboSeq data from many experiments back them up. While the complete disentangling from the external features was not feasible, this dependence stayed minimal, compared to, e.g. the work of McGillivray *et al.*, requiring eighty-nine features for classification.

To our surprise, the widely adopted across various NLP applications practice of transfer learning, where the model is pretrained on a masked language modeling objective and then fine-tuned on downstream tasks, yielded ambiguous results. DNABERT model (58) demonstrated extreme overfitting and a decrease in performance compared to DistilBert-based architecture with four times less trainable parameters: with or without pretraining the latter. We initially assumed the observation was due to us excising intronic sequences, thereby changing the native DNA structure DNABERT was trained on. Following this, we hypothesized that two rounds of fine-tuning may solve the problem: (i) MLM objective on 5'UTR sequences and (ii) token-classification objective. While DNABERT was efficient in MLM, this was not consequential for its ability to discriminate between TIS.

The resulting best model was XGBoost trained to predict the class of the start codon at the center of the sequence. It relied on experimental RiboSeq signal and employed transcript-level features—standardized per-cell expression levels. As the current model's applicability domain is limited, its primary function is to extrapolate the manually curated dataset to the other genes of the human genome. Our inference yielded that 2.18% TISs among the 5′UTRs of the 7836 of the 15 066 genes that did not undergo manual curation are likely active. While this does not close up the human uORFome annotation problem, it nevertheless provides an extensive amount of data for further hypothesis testing by other researchers. Moreover, as we demonstrated in this work, the built model has no competition, thus constituting the most reliable source of TIS predictions.

## DATA AVAILABILITY

The code and the trained model for prediction translation start sites are available via the repository https://doi.org/10.5281/zenodo.7430601.

*uORF Annotator* and up-to-date list of manually annotated ORFs in BED format are publicly available at https://doi.org/10.5281/zenodo.7435228.

## Supplementary Material

gkac1247_Supplemental_Files
