# Supplementary material for: Annotation of uORFs in the OMIM genes allows to reveal pathogenic variants in 5′UTRs
Source: Nucleic Acids Res. 2023 Jan 18;51(3):1229–44. doi: 10.1093/nar/gkac1247 (PMC9943669; doi:10.1093/nar/gkac1247)
Supplement: gkac1247_Supplemental_Files [file gkac1247_supplemental_files.zip › Figures S3-S14. Algorithm of annotation TISs in 5’UTRs using GWIPS- and Trips-browser.pptx]

## Slide 1
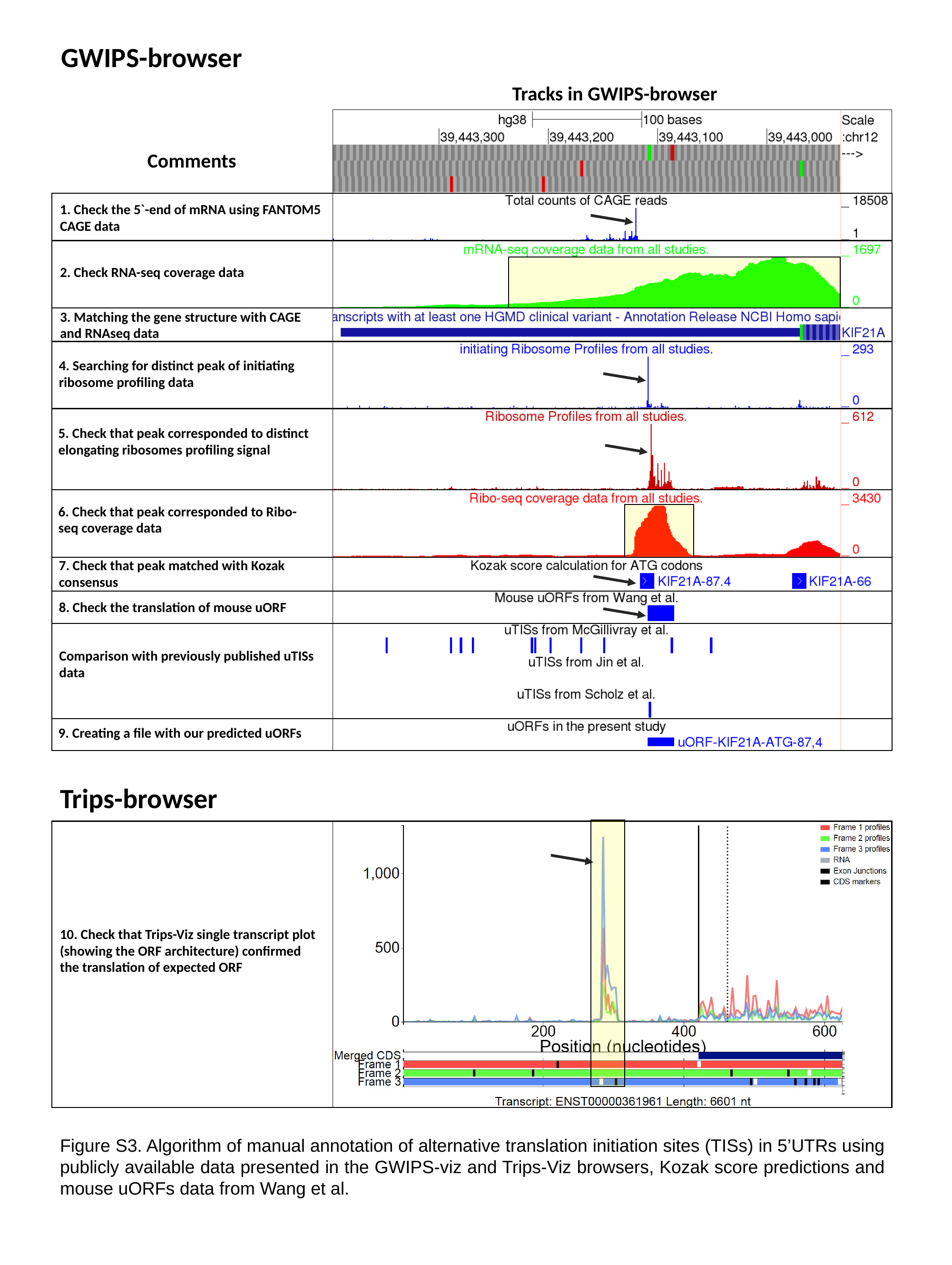

GWIPS-browser
Tracks in GWIPS-browser
Comments
1. Check the 5`-end of mRNA using FANTOM5 CAGE data
2. Check RNA-seq coverage data
3. Matching the gene structure with CAGE and RNAseq data
4. Searching for distinct peak of initiating ribosome profiling data
5. Check that peak corresponded to distinct elongating ribosomes profiling signal
6. Check that peak corresponded to Ribo-seq coverage data
7. Check that peak matched with Kozak consensus
8. Check the translation of mouse uORF
Comparison with previously published uTISs data
9. Creating a file with our predicted uORFs
Trips-browser
10. Check that Trips-Viz single transcript plot (showing the ORF architecture) confirmed the translation of expected ORF
Figure S3. Algorithm of manual annotation of alternative translation initiation sites (TISs) in 5’UTRs using publicly available data presented in the GWIPS-viz and Trips-Viz browsers, Kozak score predictions and mouse uORFs data from Wang et al.

## Slide 2
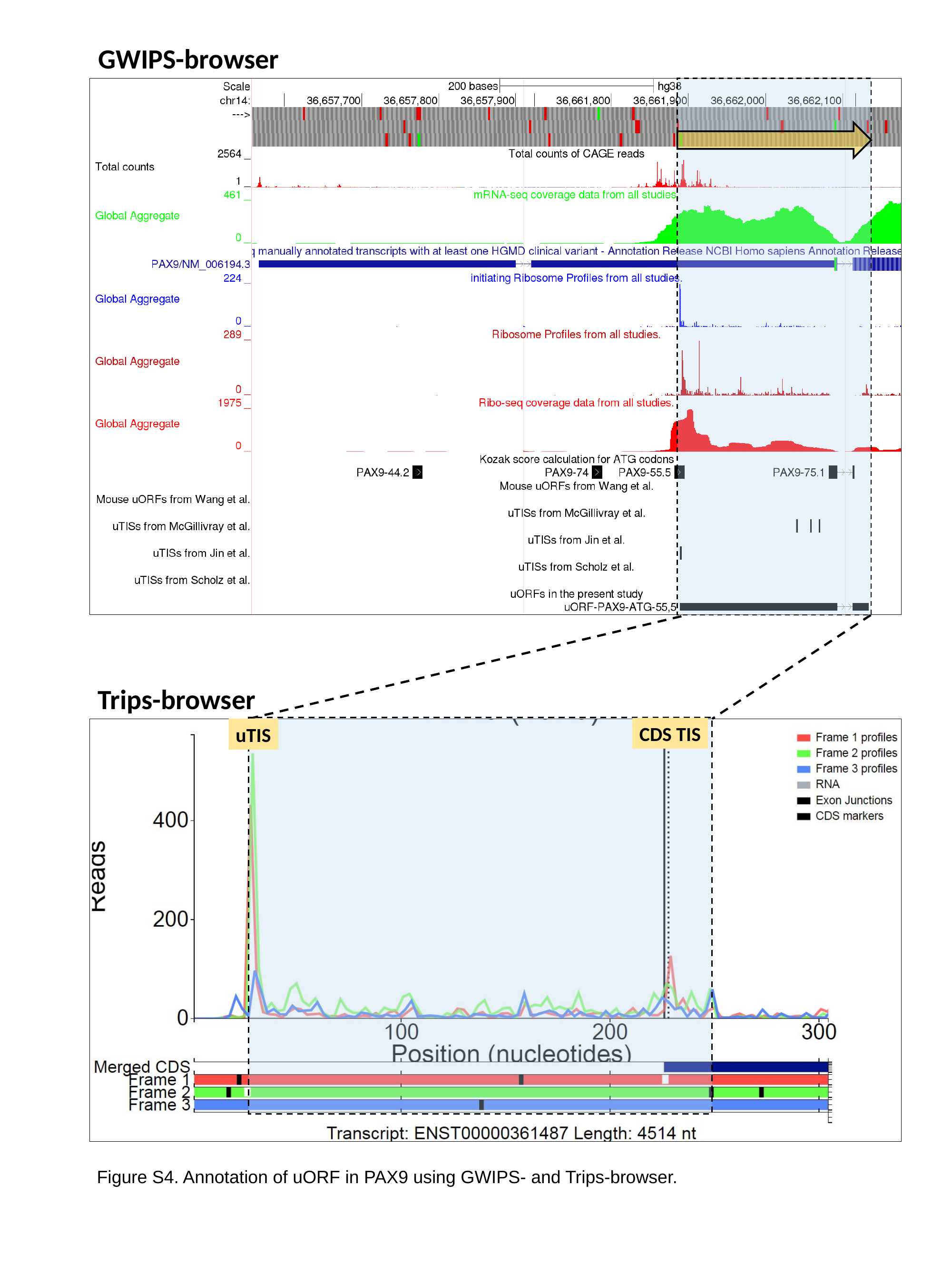

GWIPS-browser
Trips-browser
CDS TIS
uTIS
Figure S4. Annotation of uORF in PAX9 using GWIPS- and Trips-browser.

## Slide 3
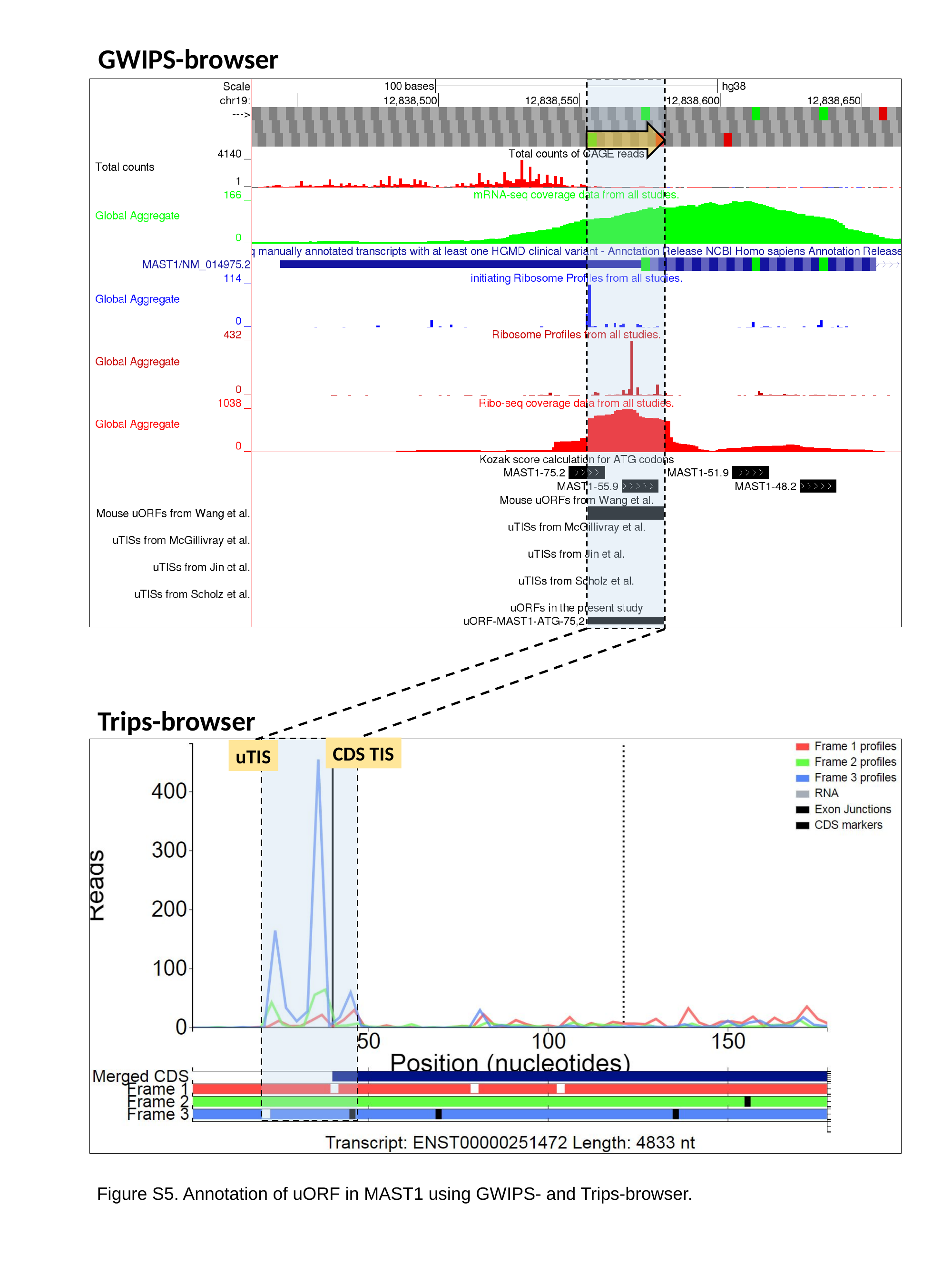

GWIPS-browser
Trips-browser
CDS TIS
uTIS
Figure S5. Annotation of uORF in MAST1 using GWIPS- and Trips-browser.

## Slide 4
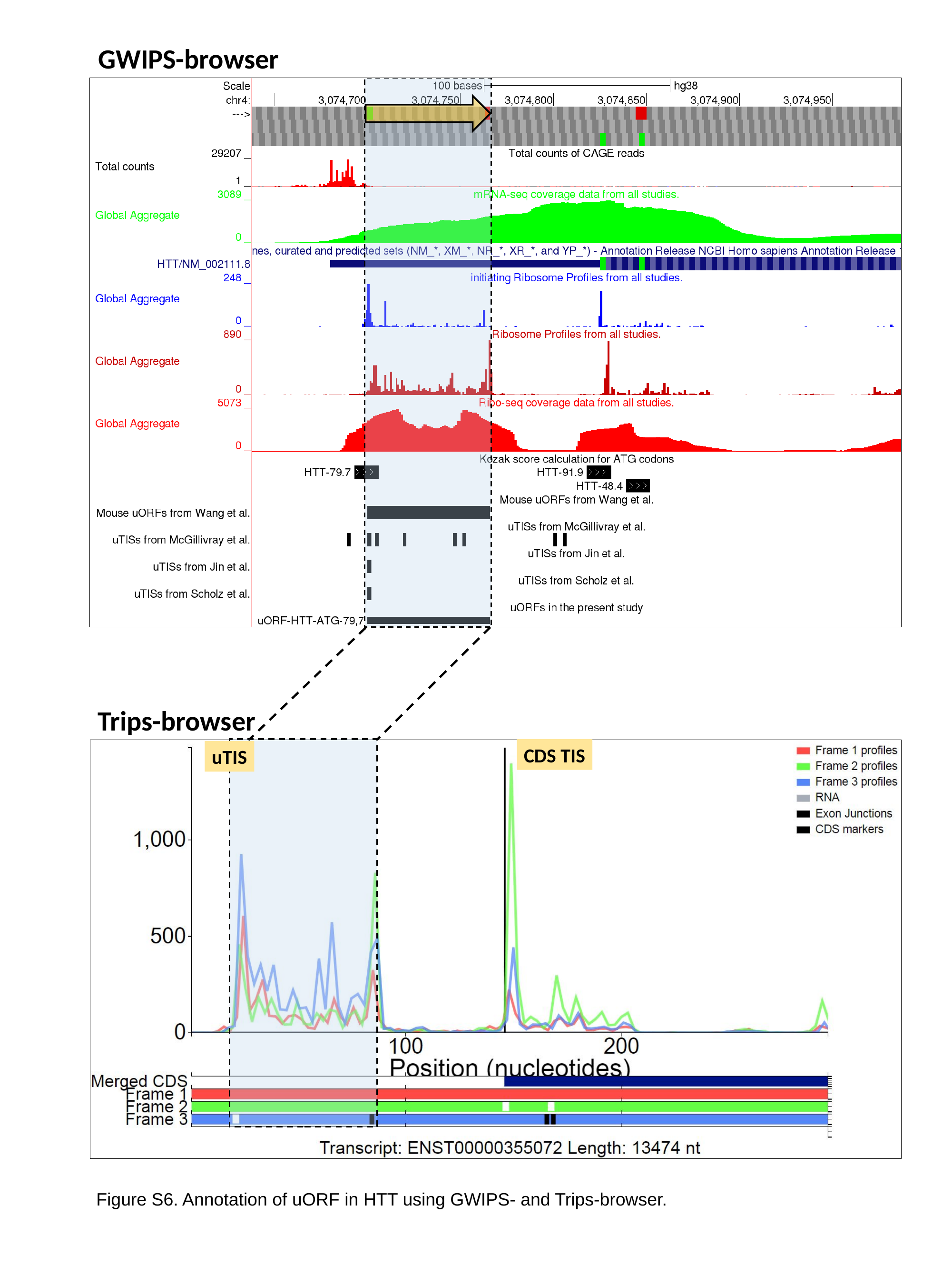

GWIPS-browser
Trips-browser
CDS TIS
uTIS
Figure S6. Annotation of uORF in HTT using GWIPS- and Trips-browser.

## Slide 5
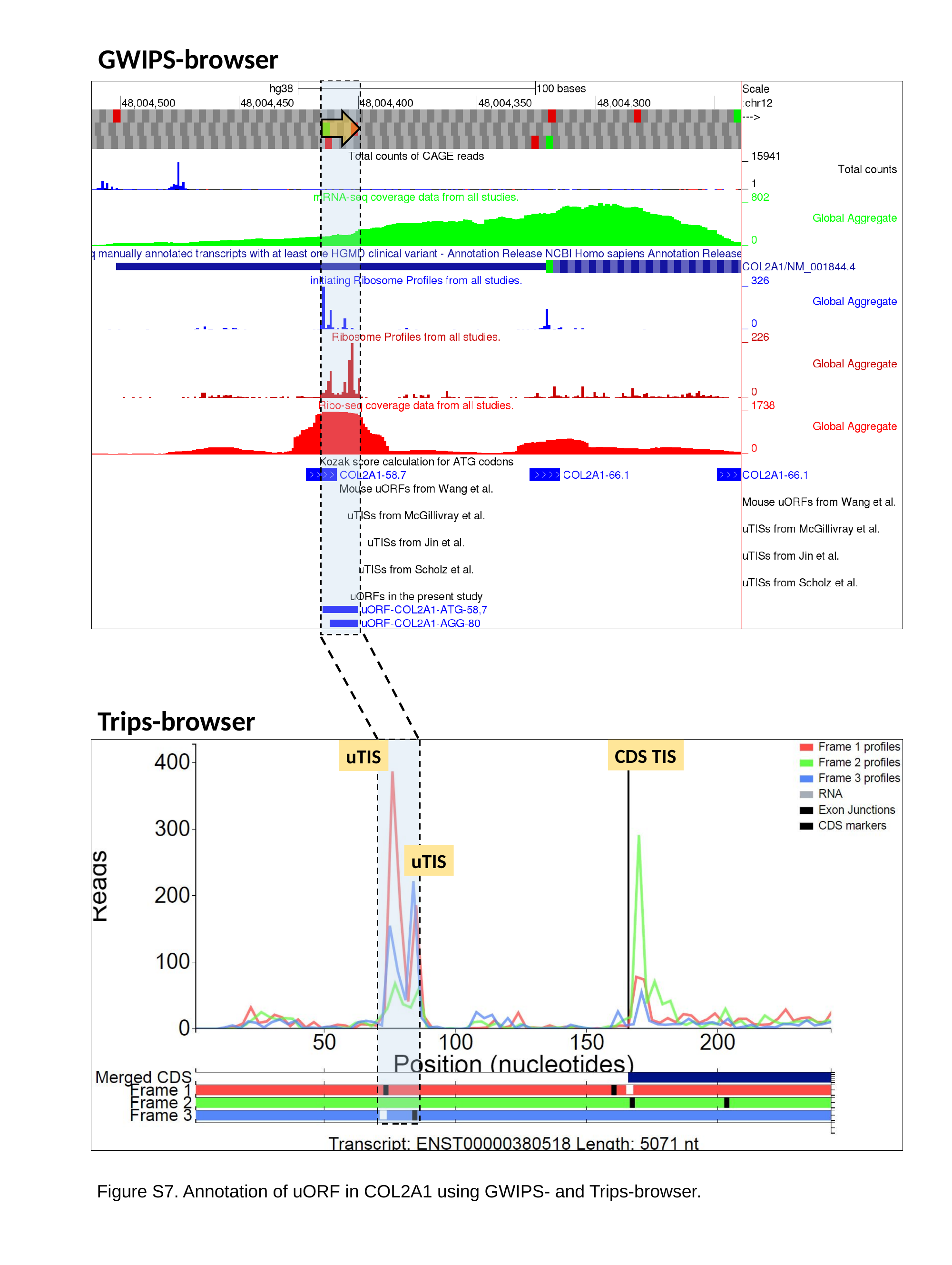

GWIPS-browser
Trips-browser
CDS TIS
uTIS
uTIS
Figure S7. Annotation of uORF in COL2A1 using GWIPS- and Trips-browser.

## Slide 6
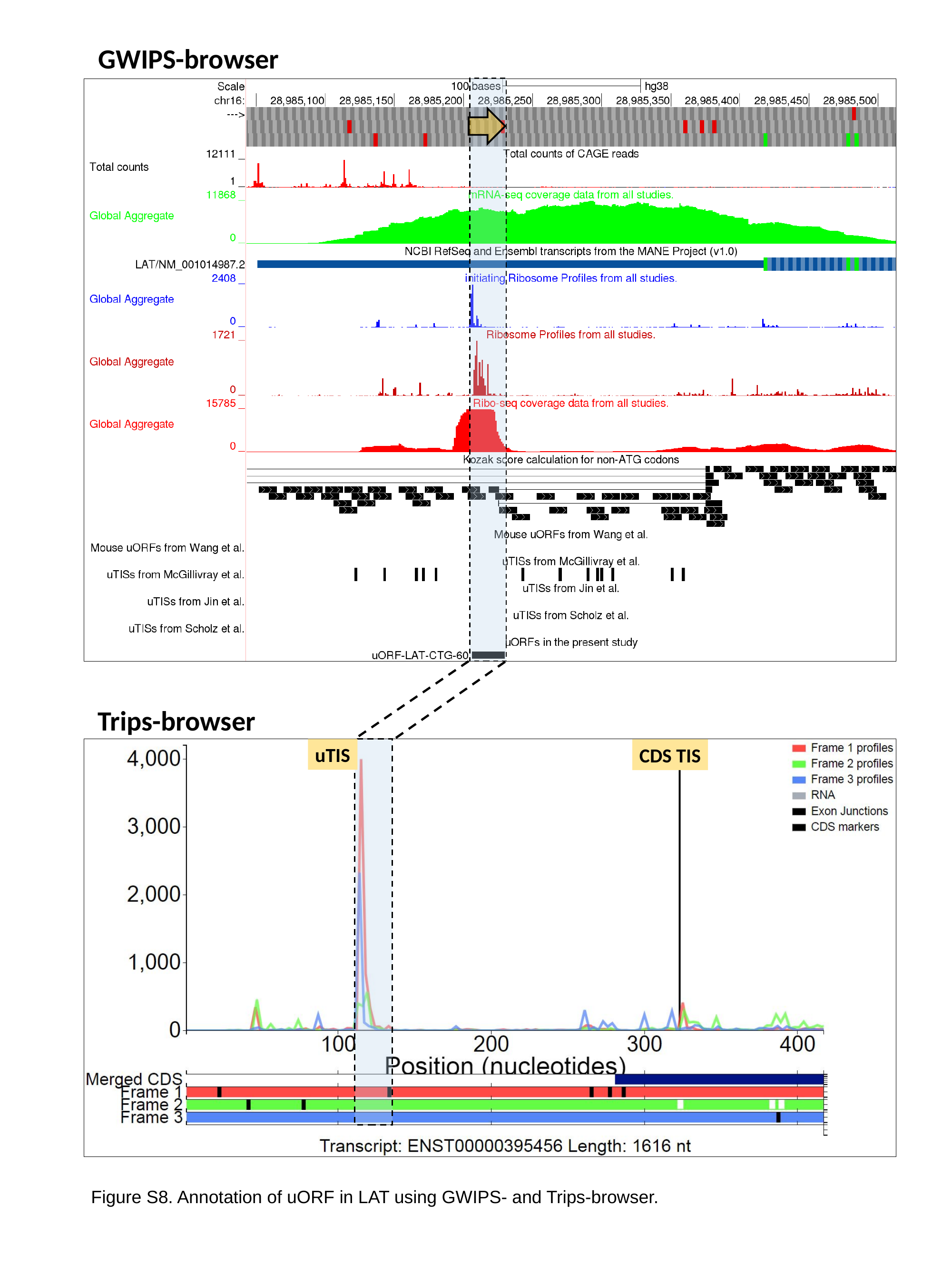

GWIPS-browser
Trips-browser
uTIS
CDS TIS
Figure S8. Annotation of uORF in LAT using GWIPS- and Trips-browser.

## Slide 7
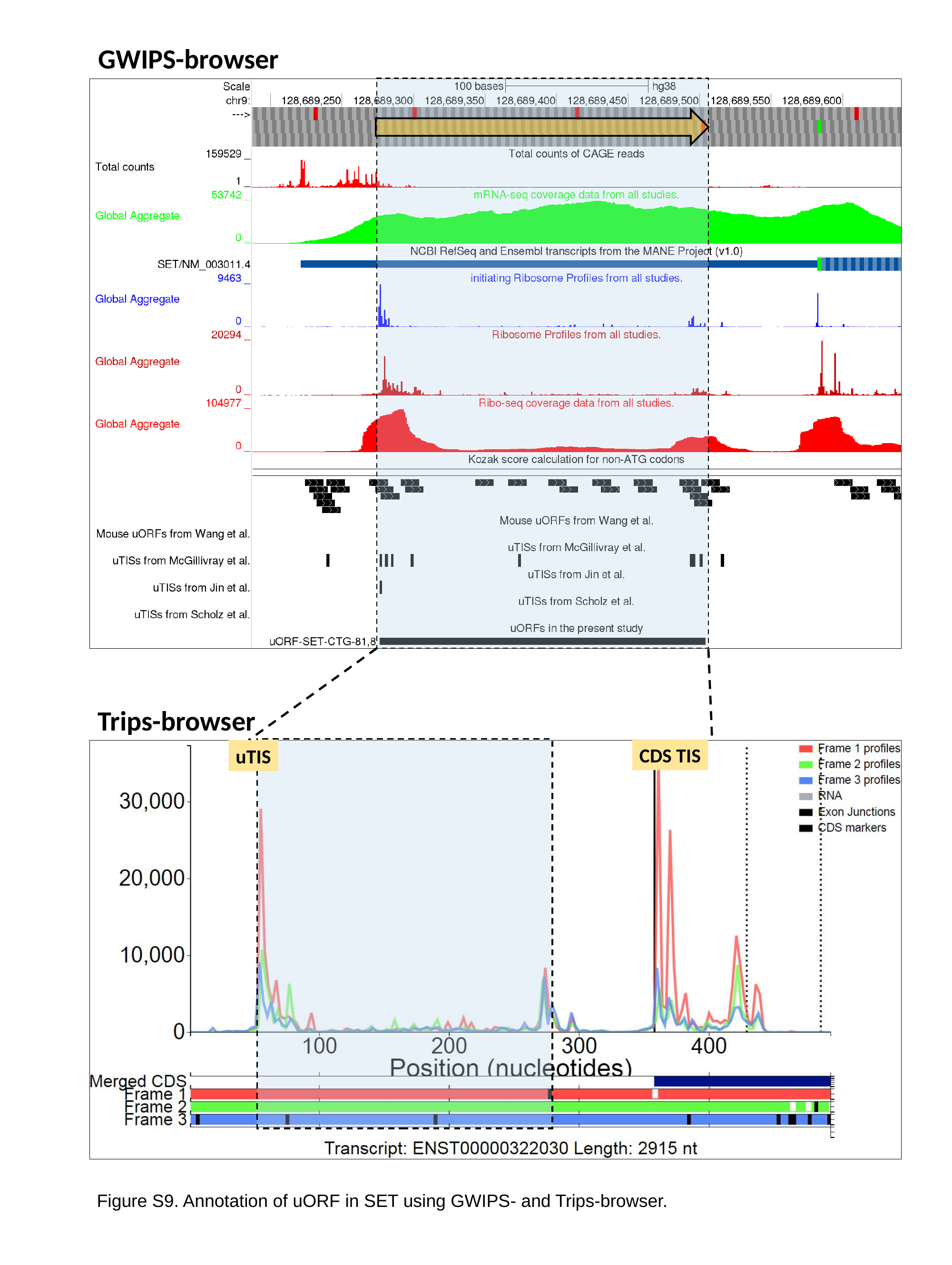

GWIPS-browser
Trips-browser
CDS TIS
uTIS
Figure S9. Annotation of uORF in SET using GWIPS- and Trips-browser.

## Slide 8
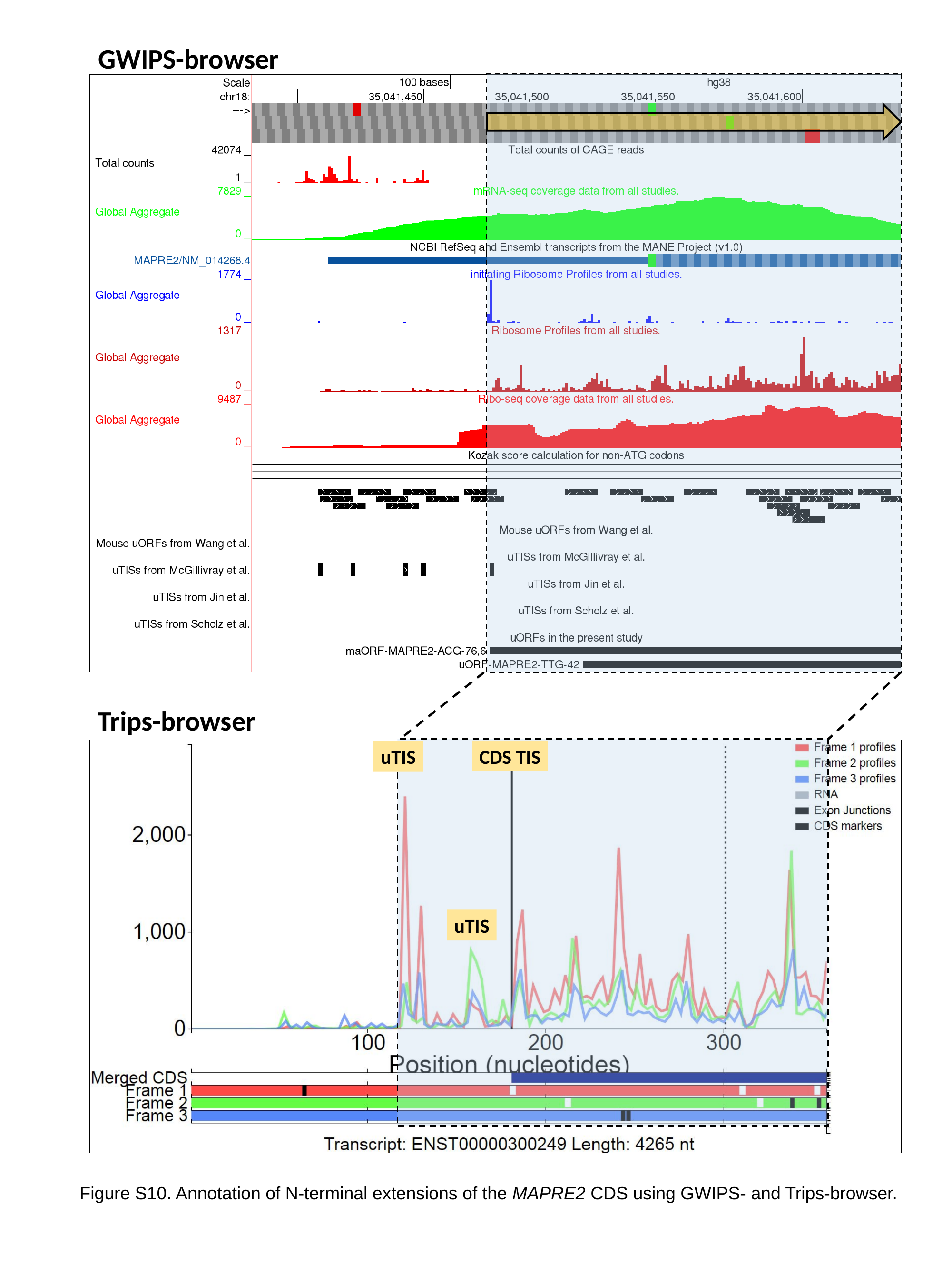

GWIPS-browser
Trips-browser
CDS TIS
uTIS
uTIS
Figure S10. Annotation of N-terminal extensions of the MAPRE2 CDS using GWIPS- and Trips-browser.

## Slide 9
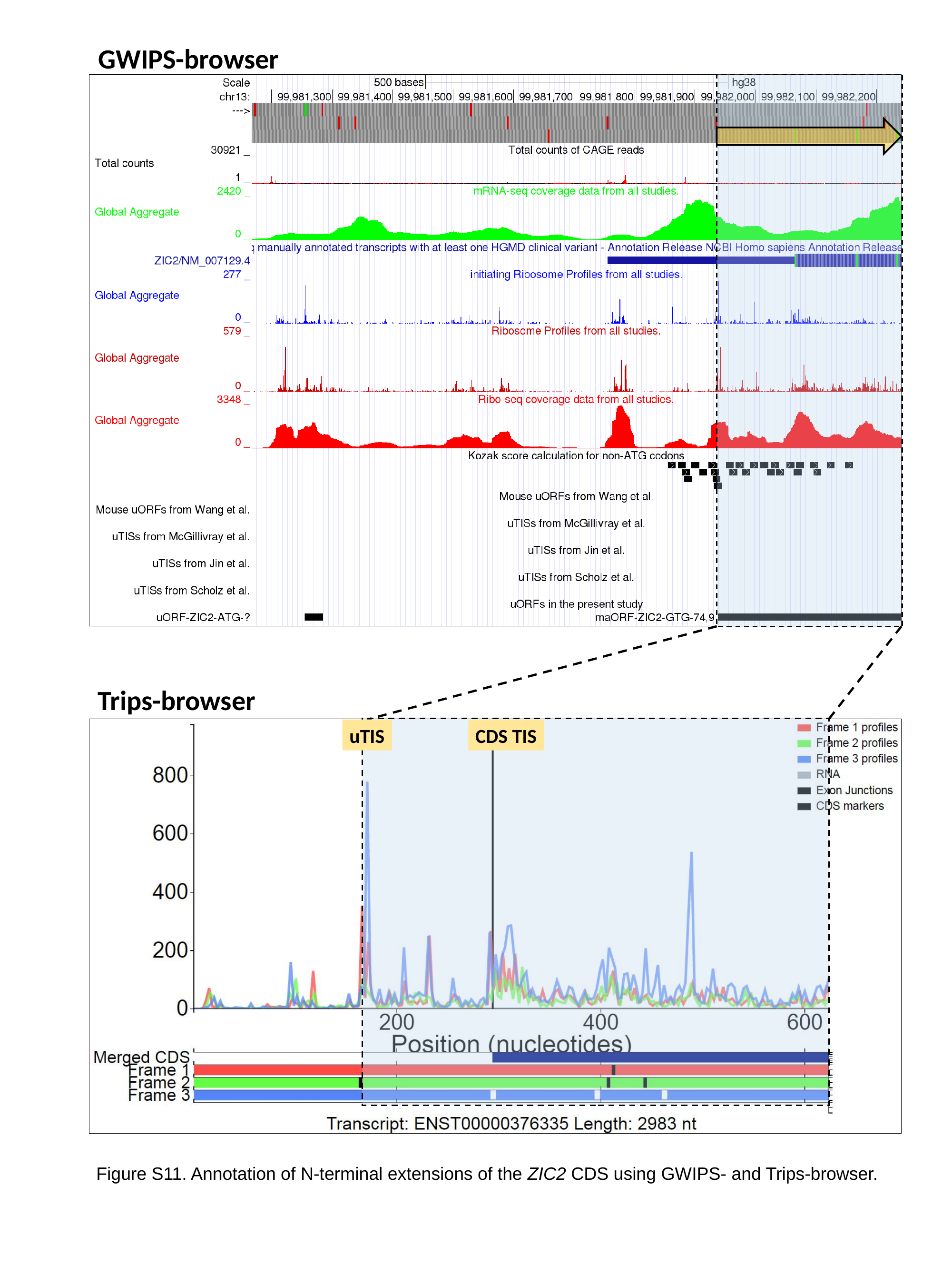

GWIPS-browser
Trips-browser
uTIS
CDS TIS
Figure S11. Annotation of N-terminal extensions of the ZIC2 CDS using GWIPS- and Trips-browser.

## Slide 10
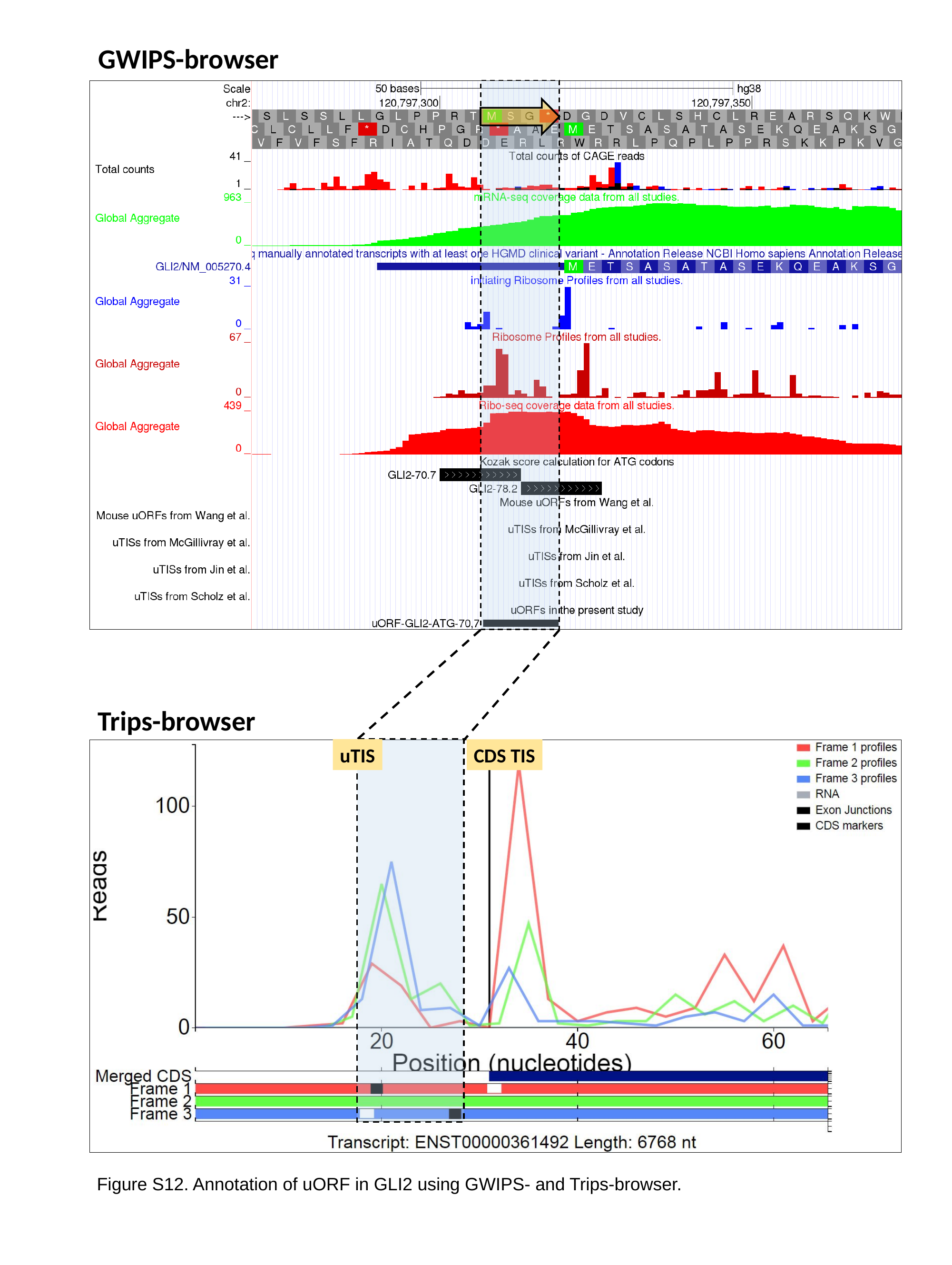

GWIPS-browser
Trips-browser
uTIS
CDS TIS
Figure S12. Annotation of uORF in GLI2 using GWIPS- and Trips-browser.

## Slide 11
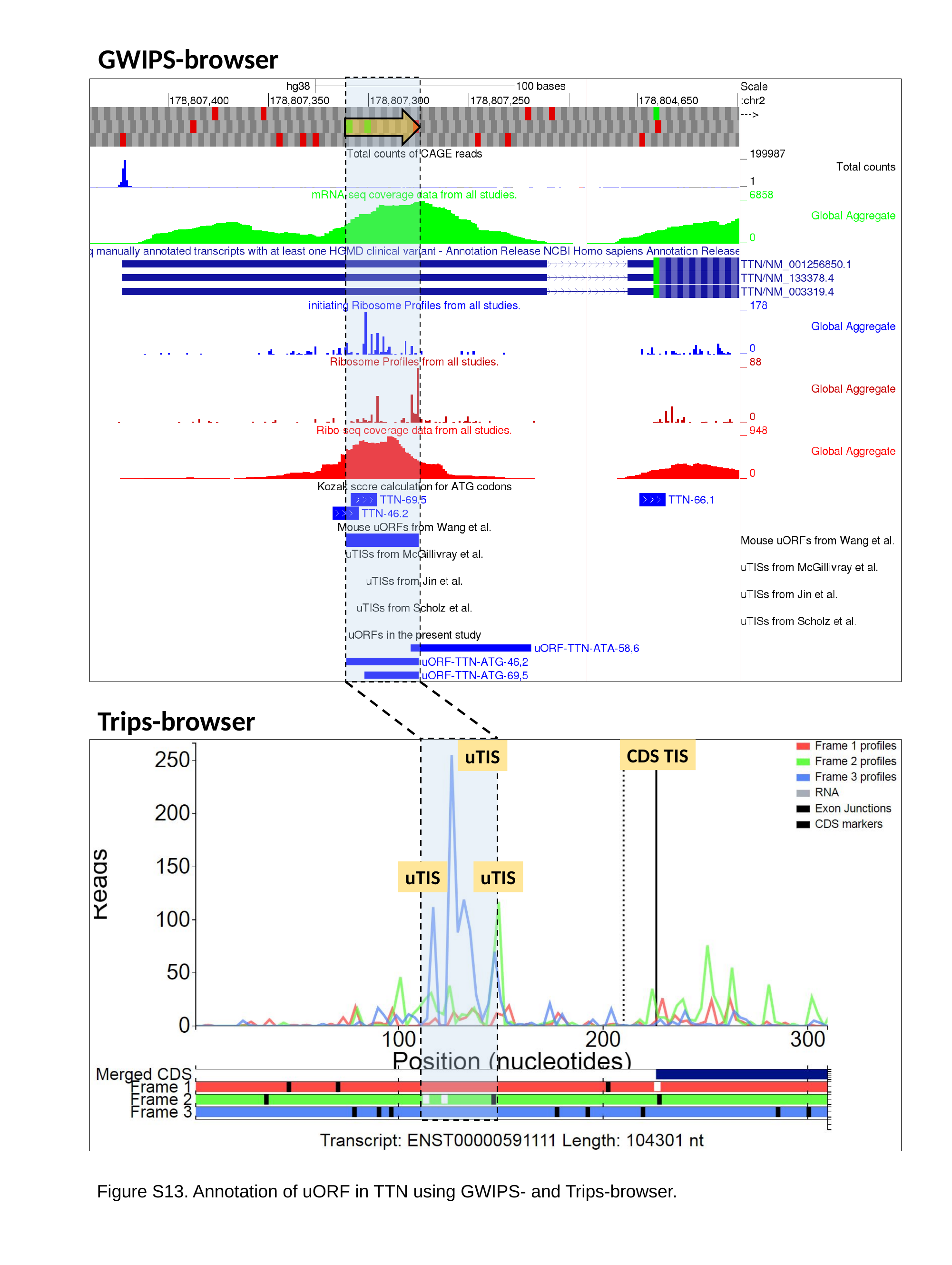

GWIPS-browser
Trips-browser
CDS TIS
uTIS
uTIS
uTIS
Figure S13. Annotation of uORF in TTN using GWIPS- and Trips-browser.

## Slide 12
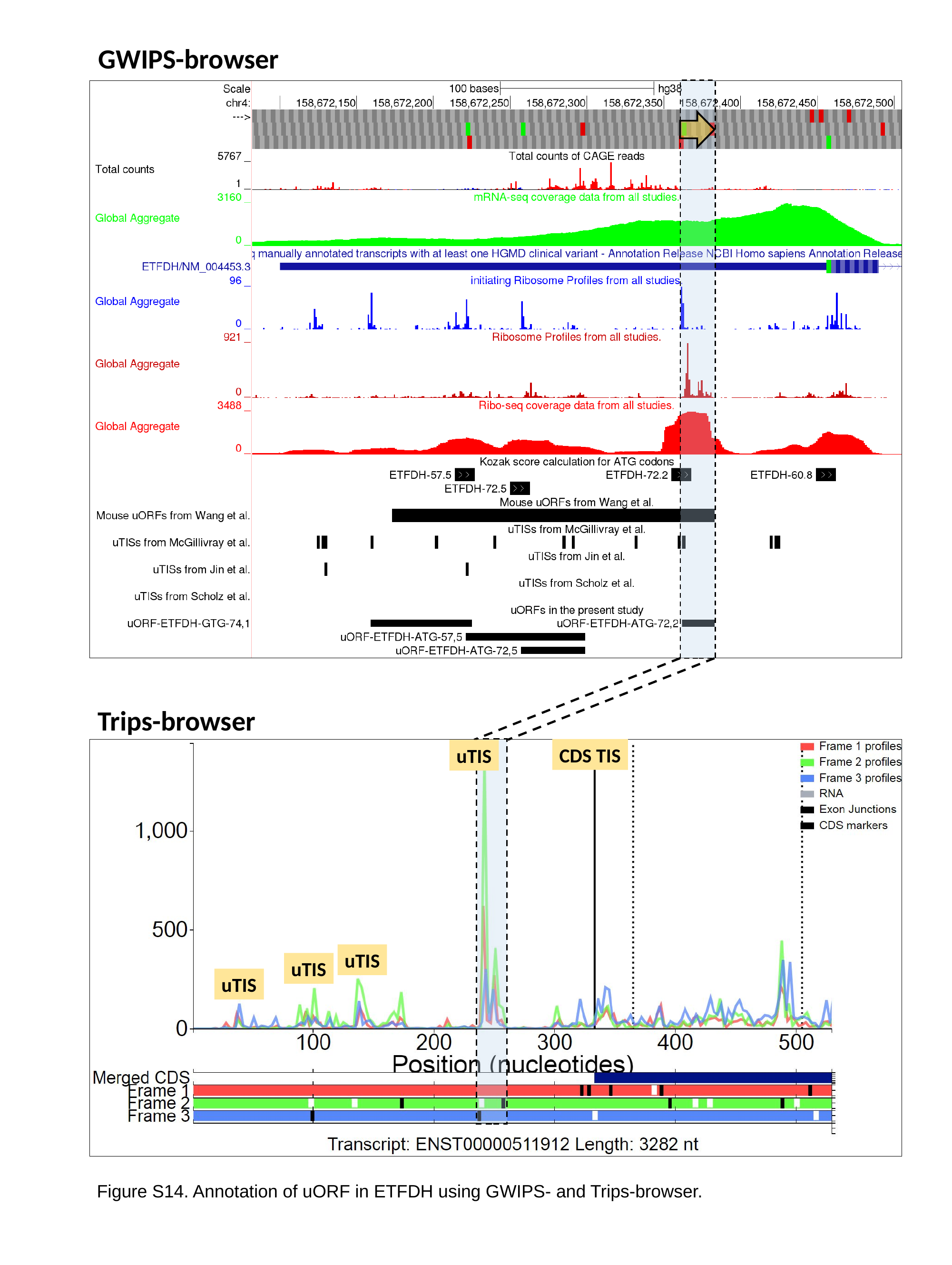

GWIPS-browser
Trips-browser
CDS TIS
uTIS
uTIS
uTIS
uTIS
Figure S14. Annotation of uORF in ETFDH using GWIPS- and Trips-browser.
