# Supplementary material for: Annotation of uORFs in the OMIM genes allows to reveal pathogenic variants in 5′UTRs
Source: Nucleic Acids Res. 2023 Jan 18;51(3):1229–44. doi: 10.1093/nar/gkac1247 (PMC9943669; doi:10.1093/nar/gkac1247)
Supplement: gkac1247_Supplemental_Files [file gkac1247_supplemental_files.zip › Supplementary Methods.pdf]

Supplementary Materials and Methods

ML learning objective and models' architectures

Generally, we sought a sequence-based classifier annotating putative TISs. In an ideal scenario, it would depend on no hand-crafted features other than the primary sequence itself, thereby functionally mimicking a ribosome. A recent success in natural language modeling tasks found its way into biological sequence modeling. Their unifying framework is masked language modeling (MLM), where a pre-training to fill the gaps in the supplied text yields a general-purpose model capturing natural interdependencies within the input. Subsequently, fine-tuning the model on specific downstream objectives is thought to be easier and achieve better results. This two-stage process constitutes the core of the transfer learning paradigm.

An example of such a model working on DNA sequences is the DNABERT. During the MLM stage, it splits the DNA sequence into successive k-mers, each constituting a separate language unit, or token. It then masks a random 15% of the input tokens and attempts to recover them using the surrounding context. A pre-trained model is saved and fine-tuned for specific objectives, such as splice-site or SNP effect prediction.

In this work, we fine-tuned the pre-trained DNABERT model to annotate TISs. As its performance was low, we attempted to create a similar model based on closely related distilBERT architecture that uses a smaller pool of trainable parameters (1). In both cases, architectures encompass BERT — bidirectional encoder representations from transformers (2). The key principle behind BERT is the transformer architecture relying on the self-attention mechanism (3). In simple terms, for each input token, it learns contextual embeddings, or high-dimensional vector representations, by simultaneously attending to all other input tokens via a set of matrix multiplications. For a more detailed overview, we refer readers to original publications.

Neural networks typically utilize a vast number of trainable parameters, thereby requiring large training sets to train well. The underlying model's complexity may come as both beneficial and deleterious. In some cases, such as ours, a simpler model and learning objective may help to overcome these limitations. As a result, we used a lightweight distilBERT architecture trained on tokenized sequences and linearly scaled ribo-seq signal. We injected the latter by summing with the last dimension of the embedding layer. One can view the latter as the extension of the one-hot encoding described in the main text, where the vector values are optimized during training, and the dimension of each vector is a pre-specified parameter. In transformer models, this size is propagated into the subsequent transformer blocks and thus constitutes the sequence's internal representation size, defining the overall model's complexity. In BERT-like models, these internal embeddings are processed by multiple self-attention blocks (heads), each forming different "views" of the data. In our case, abandoning the MLM objective while using the experimental signal and reducing the model's complexity via setting a smaller number of layers - 3, embedding size - 36, and the number of self-attention heads - 3 positively impacted the performance, leading to an architecture depicted in Figure S1.

Decision tree-based ensemble models, such as Random Forest (RF), comprise a computationally cheaper alternative to NNs. In such models, a collection of weak learners works in

tandem to produce a more potent classifier. Here, we used gradient-boosted trees from the xgboost library (4). Compared to the RF, where trees are grown in parallel on random bootstraps of the training data, XGBoost adds trees sequentially, improving the overall model's performance with each added tree by implicitly optimizing the objective function — a logistic loss, in our case.

Employing a simpler model enabled two additional techniques: (1) automatic tuning of non-trainable parameters to improve the model's performance and (2) cross-validation to assess the generalization capacity better. For the former, we used the Optuna library (Table S1). During the latter, based on gene IDs, we sequentially partitioned the data into training (90%), and testing (10%) folds, yielding ten different partitions.

Performance evaluation metrics

For binary classification, we define TP and FN as the number of correctly (true positives) predicted positive and incorrectly predicted negative (false negative) instances. At the same time, TN is the number of correctly predicted negative examples, and FP is the number of incorrectly predicted positive cases (false positives). Thus, precision, PRC=TP/(TP+FP), reflects how many actual positive instances were correctly predicted among all the predicted positives. Recall, or true positive rate, REC=TP/(TP+FN) accounts for the number of correctly predicted positive instances across all positives. In turn, specificity, or true negative rate, is defined as SPC=TN/(TN+FP) and is equivalent to recall when negative and positive classes are inverted.

Composite metrics, such as the F1 score and balanced accuracy, combine the metrics defined above and are typically used for class-imbalanced datasets as opposed to regular accuracy — the fraction of correctly predicted instances. Thus, balanced accuracy is the arithmetic mean between recall and specificity, BAC=1/2(REC+SPC). It can also be interpreted as the average recall across classes. Finally, F1-score combines precision and recall via their geometric mean into F1=2*PRC*REC/(PRC+REC). It doesn't depend on TN instances, instead focusing on how well the model predicts the positive ones.

REFERENCES

1. Sanh, V., Debut, L., Chaumond, J. and Wolf, T. (2019) DistilBERT, a distilled version of BERT: smaller, faster, cheaper and lighter.
2. Devlin, J., Chang, M.-W., Lee, K. and Toutanova, K. (2018) BERT: Pre-training of Deep Bidirectional Transformers for Language Understanding.
3. Vaswani, A., Shazeer, N., Parmar, N., Uszkoreit, J., Jones, L., Gomez, A.N., Kaiser, L. and Polosukhin, I. (2017) Attention Is All You Need.
4. Chen, T. and Guestrin, C. (2016), *Proceedings of the 22nd ACM SIGKDD International Conference on Knowledge Discovery and Data Mining*. Association for Computing Machinery, San Francisco, California, USA, pp. 785–794.

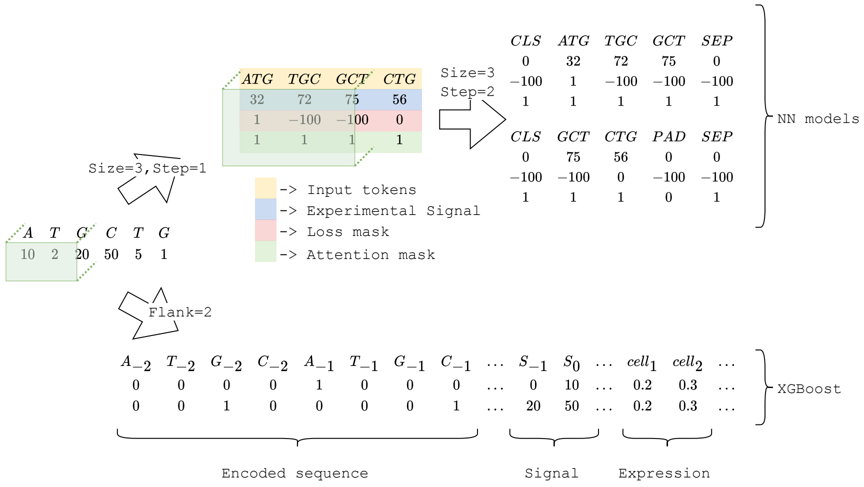

Figure S1: The demonstration of the input preparation for neural networks (top) and XGBoost (bottom) on a sample sequence ATGCTG. For NNs, we first split the sequence into successive k-mers with $k = 3$. We summed the experimental signal for each k-mer. We slid a window over the k-merized sequence using the pre-specified size and step parameters. For XGBoost, we centered the sequence at the start codon and used pre-specified flank size for neighboring characters. We encoded each character using the one-hot approach, where a character's presence/absence is denoted by one or zero. We concatenated the encoded sequence with the experimental signal and transcript-wise expression levels.

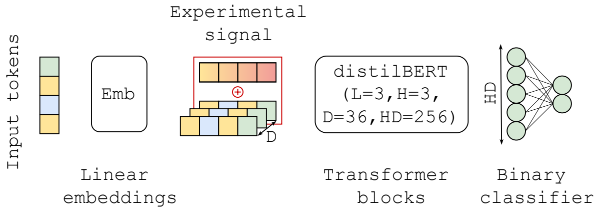

Figure S2: NN model's architecture. Input tokens – integer encoded k-mers – were embedded in a D-dimensional space with `Embedding` layer. Following this, the experimental signal over the k-merized sequence is injected into the model by summing with the last embedding dimension. The resulting embedded sequence – a matrix of $S \times D$, where S is the sequence's length, is fed into three lightweight distilBERT blocks (3 heads and the dimension of the hidden feed-forward layer of 256) that return the matrix with the exact dimensions. The latter is processed by a single feed-forward layer outputting probabilities of classes 0 and 1.

Parameter	Min	Max	Result	Description
learning_rate	10^{-2}	2	0.1	The step in the gradient descent algorithm.
max_depth	4	20	8	Maximum tree depth.
gamma	10^{-2}	2	0.32	Regularization parameter – a complexity cost associated with introducing nodes.
reg_lambda	0	20	2.20	Weight of the l_2 regularization.
reg_alpha	0	20	17.90	Weight of the l_1 regularization
colsample_bytree	0.1	1.0	0.59	A fraction of features sampled for an individual tree.
colsample_bylevel	0.1	1.0	0.73	A fraction of features sampled when introducing a node of an individual tree.
scale_pos_weight	1	20	10.65	For binary classification, the loss multiplier for 1-labeled instances.

Table S1: XGBoost's hyperparameters subject to optimization and the resulting values.

	# Positive	# Negative	F_1	PRC	REC
CTG	1612	53707	0.66	0.55	0.82
ATG	1603	18784	0.66	0.61	0.74
GTG	648	35139	0.62	0.50	0.83
ACG	307	12834	0.63	0.52	0.80
TTG	292	28353	0.55	0.44	0.77
ATC	157	18633	0.58	0.49	0.74
ATT	152	22101	0.53	0.42	0.74
ATA	47	13107	0.46	0.37	0.63
AGG	38	47860	0.24	0.28	0.24
AAG	15	28818	0.06	0.12	0.04

Table S2: Ten-fold cross-validated performance of the XGBoost model.

Primer	Sequence (5` -> 3`)	Purpose
VecF	TGGCTTCCAAGGTGTACGACC	Vector amplification
VecR	TGTTGTGTCAGAAGAATCAAGCTTTTTGCAA	
MAPRE2F	AAAAGCTTGATTCTTCTGACACAACAGAGAAGGCAGTGAGCGAGCA	for cloning
MAPRE2R	GTCGTACACCTTGGAAGCCAACAGGGTTTGGGTCGGC	for cloning
ZIC2F	AAAAGCTTGATTCTTCTGACACAACAGGACTCTTCTCCTCCTCCACC	for cloning
ZIC2R	GTCGTACACCTTGGAAGCCAACTGCGGACCCGCG	for cloning
HTTF	AAAAGCTTGATTCTTCTGACACAACAGCTGCCGGGACGGGTC	for cloning
HTTR	GTCGTACACCTTGGAAGCCAAGGCCTTCATCAGCTTTTCCAG	for cloning
COL2A1F	AAAAGCTTGATTCTTCTGACACAACAGCAGAGCGCTGCTGG	for cloning
COL2A1R	GTCGTACACCTTGGAAGCCAACGTCTGGGGAGCCCCGA	for cloning
LATF	AAAAGCTTGATTCTTCTGACACAACAACAGCTTCCTGCCGCAG	for cloning
LATR	GTCGTACACCTTGGAAGCCAAGGGGACCAGGATGGCCTC	for cloning
SETF	AAAAGCTTGATTCTTCTGACACAACAGCCGTAGGAGGAGGTGGA	for cloning
SETR	GTCGTACACCTTGGAAGCCAAGACTTTGGCCGCCG	for cloning
PAX9F	AAAAGCTTGATTCTTCTGACACAACAAGCCCACGTTGCTGCTT	for cloning
PAX9R	GTCGTACACCTTGGAAGCCAACTCCATTGCTCCGAGCAG	for cloning
MAST1F	AAAAGCTTGATTCTTCTGACACAACAGCCGCCGCCTCCG	for cloning
MAST1R	GTCGTACACCTTGGAAGCCAAGAGAGAGTCAGACATGACCCG	for cloning
TTNvecF	CTAGAAAGATGACAACTCAAGCACCGACGTTGGCTTCCAAGGTGTACGACC	Vector amplification for TTN cloning
TTNvecR	GAATGCACGACTGCTCTGTTGTGTCAGAAGAATCAAGC	
TTNins1F	GAGCAGTCGTGCATTCCCAG	for cloning
TTNIns12R	GCTTGAGTTGTCATCTTTCTAGGCACTCTGATTTCTCAAGAGTGCCTAAAAAGG	for cloning
GLIvecF	CCAGGACGATGAGCGGCTGAGATGGAGACGTTGGCTTCCAAGGTGTACGACC	Vector amplification for GLI2 cloning
GLIvecR	AGTCCACGAACTTCACTTGTTGTGTCAGAAGAATCAAGC	
GLIIns1F	AGTGAAGTTCGTGGACTCCTAC	for cloning
GLIIns12R	CGCTCATCGTCCTGGGTGGCAATCCTTGCTCTTTGATGTGTCGGTAAAG	for cloning
ETFDHvecF	CCGCTAGCCAAGCTGTTGGCTTCCAAGGTGTACGACC	Vector amplification for ETFDH cloning
ETFDHvecR	GACCGCTCTTGCTTCACTGTTGTGTCAGAAGAATCAAGC	
ETFDHF	GTGAAGCAAGAGCGGTCGG	for cloning
ETFDHR	ACAGCTTGGCTAGCGGC	for cloning
MAPRErefF	CTTCTCGGGAGTGCGCCACCTGGGCCGACCCAAACC	for mutagenesis
MAPREpredF	AGGCGAGCGAGCGGGAAGCAGCCACCTTCCTCACCAGC	for mutagenesis
ZIC2refF	GGCGGGCGCGCTGGCCCTCCTGGACGCGGGTCCG	for mutagenesis
ZIC2predF	GCGGCTCCCAGGGCTGAAGCCGCCACCACCGCCG	for mutagenesis
HTT-SRF	CATTGCCCCGGTGCTGGCGGCGCCGCGAGTCG	for mutagenesis
HTT_ATdelF	TGCCGGGACGGGTCCAAGGACGGCCGCTCAGGTTCT	for mutagenesis
COL2A1SRR	GCGGGTCCGGGTCTGTACCGCGCCCTCATGCAG	for mutagenesis

COL_ATdelF	CAAGGGCCTCCTGCAGGGCGCGGTACAGAC	for mutagenesis
LAT-SRR	GGATGGGTCCCCTCTTTCTGGGCTGAGTGTGCGTC	for mutagenesis
LAT_stdelF	GGACTTTCCACAGTCAGGACGCACACTCAGCCCAG	for mutagenesis
SET-SRF	CCGCGCGTGTGGCGTGGGGGAAGCCGCTTGCC	for mutagenesis
PAX9atgdelF	CCACGTTGCTGCTTAGATTGAACAGAACTCAAGCCTCTTTCATC	for mutagenesis
SET_stdelF	GAGAGCGAGCAGCGAGGCTGGATCGCCGAGCGC	for mutagenesis
MASTatgdelR	ACCCGGCGGCGGCGAGGTGGCAGGTGCGGCA	for mutagenesis
TTNmutF1	CAGAAAAACCAACTCTCCATAcGACGTCGTTTCAGAAGC	for mutagenesis
TTNmutR2	TGGAGAGTTGGTTTTTCTGACCTCCGAGAATCGGTGAGCCTCTAATCCT AA	for mutagenesis
GLImutF1	CCAGGACGATGAGCGGCTGCGATGGAGACGTTGGCTTCCAA	for mutagenesis
GLImutR2	GCCAACGTCTCCATCGCAGCCGCTCAaCGTCCTGGGTGGCAATC	for mutagenesis
ETFDHmut1F	CGCCCCCCGCGGCCTGGAGGTCCAGCGCCCG	for mutagenesis
ETFDHMut2R	GCGGGGGGCGCCACCTGCCGGAAAGCAAGAAC	for mutagenesis

Table S3. The primers used for cloning and mutagenesis.
